# Mental and physical health of US rural/urban caregivers of persons with dementia

**DOI:** 10.1371/journal.pone.0329260

**Published:** 2025-08-01

**Authors:** Phoebe Tran, Fei Wang, E-Shien Chang

**Affiliations:** 1 Department of Public Health, University of Tennessee, Knoxville, Tennessee, United States of America; 2 College of Social Work, University of Tennessee, Knoxville, Tennessee, United States of America; 3 Division of Geriatrics and Palliative Medicine, Weill Cornell Medical College, New York City, New York, United States of America; University of Bamberg, GERMANY

## Abstract

**Purpose:**

Increased dependence on caregiving and limited access to healthcare services in rural US communities may contribute to worse mental and physical health in rural caregivers of persons with dementia (PWD) relative to their urban counterparts. We assessed the association between rural/urban residence and mental and physical health among US caregivers of PWD.

**Methods:**

Using 2020–2022 Behavioral Risk Factor Surveillance System data, we identified caregivers of PWD from rural (n = 2311) and urban (n = 15094) areas. Mental health outcome was operationalized as poor mental health days (PMHD); categorized as 0, 1–13, and ≥14 PMHD in previous month. Poor physical health (PPHD) was operationalized in the same manner. Covariates included socio-demographic and caregiving factors. Four sets of unadjusted and adjusted survey-weighted multinomial logistic models (reference: 0 days) were created for PMHD and PPHD.

**Results:**

Approximately, 25.7% of rural and 20.8% of urban caregivers reported ≥14 PMHD while 25% of rural and 10% of urban caregivers reported ≥14 PPHD. Prior to adjustment, rural caregivers had lower odds (0.59, 95% CI: 0.34–1.05) of 1–13 vs. 0 PMHD but higher odds (1.13, 95% CI: 0.55–2.30) of 14 + vs 0 PMHD compared to urban caregivers with neither association being statistically significant. In adjusted models, the association for 1–13 vs 0 PMHD became significant, while rural residence became associated with lower, non-significant odds of 14 + vs. 0 PMHD. For physical health, rural caregivers had lower odds (0.86, 95% CI: 0.53–1.41) of 1–13 vs 0 PPHD but higher odds (2.57, 95% CI: 1.00–6.63) of 14 + vs 0 PPHD in unadjusted models with neither result being significant. After adjustment, the associations for 1–13 vs. 0 PPHD were attenuated and remained non-significant, while rural caregivers had significantly higher odds of 14 + vs 0 PPHD, consistent with unadjusted results.

**Conclusions:**

Rural caregivers of PWD are less likely to experience short-term mental health problems compared to their urban counterparts. However, they face similar levels of experiencing PMHD. Additionally, rural caregivers of PWD are more likely to endure more PPHD than urban caregivers. Considering the extensive day-to-day responsibilities that caregivers of PWD carry and the ongoing need for their support, it is crucial to enhance long-term mental health resources for both rural and urban caregivers. Furthermore, targeted initiatives to support the long-term physical health of rural caregivers are equally essential.

## Introduction

An estimated 22.3% of US adults serve as caregivers, defined as individuals who provide care to family members or friends usually without pay [1]. With the rapid aging of the US population, the need for caregivers is expected to rise in the coming decades [2]. This gap is particularly pronounced for persons with dementia (PWD), who generally require high levels of care as the illness progresses. It has been estimated that more than 75% of PWD receive their day-to-day support and care from caregivers [3]. Caregiving duties for PWD can include assisting with scheduling and transportation to medical appointments and activities of daily living (i.e., feeding, dressing, personal hygiene) [4,5]. Despite known positive aspects of caregiving for PWD, there is substantial evidence indicating that caregivers experience poorer mental and physical health outcomes than individuals who do not serve as caregivers [6,7].

The higher reliance on caregiving and lower availability of healthcare services for both the caregiver and the person receiving care in rural US communities may contribute to caregivers in US rural areas having worse mental and physical health than those in urban areas [8]. Existing US research examining differences in mental and physical health between US rural and urban caregivers of PWD has yielded inconsistent results [9,10]. These varied results may be attributed to prior studies being conducted in a single state, relying on conveniently sampled data, considering either mental health only, and using an outcome that does not distinguish between mental and physical health [9,10]. Information from across the US on comparisons of the mental and physical health of US rural and urban caregivers of PWD could help inform coordinated efforts in allocation of mental and physical health resources tailored toward the unique strengths, needs, and stressors among these diverse caregiver groups [11].

Our study sought to examine if poor mental health days (PMHD) and poor physical health days (PPHD) differed between US rural and urban caregivers. The association between rural/urban residence and PMHD and PPHD was assessed prior to and following adjustment for health-related sociodemographic and caregiving characteristics. Owing to limited healthcare services in rural areas, we hypothesized that rural caregivers of PWD would have a greater number of PMHD and PPHD than their urban counterparts.

## Materials and methods

### Study participants

We used the 2020–2022 Behavioral Risk Factor Surveillance System surveys (BRFSS) for this study. The BRFSS is a publicly available nationally representative annual survey administered via mobile phone and landline by the Centers for Disease Control and Prevention (CDC) to noninstitutionalized individuals aged ≥18 years in all 50 US states, Washington D.C., and the US territories [12]. The survey contains information on participants’ sociodemographic background, health conditions, and healthcare utilization [12]. By applying survey weighting and oversampling of underrepresented groups, the CDC ensures that analyses using BRFSS data are representative of the general US population [12]. All BRFSS data used for this paper is publicly available at the CDC’s website: https://www.cdc.gov/brfss/index.html.

From the BRFSS data, we identified caregivers of PWD as individuals having a “Yes” response to the BRFSS question, “During the past 30 days, did you provide regular care or assistance to a friend or family member who has a health problem or disability?” and a “Alzheimer ´s disease, dementia or other cognitive impairment disorder” response to the BRFSS question “What is the main health problem, long-term illness, or disability that the person you care for has?” [13]. Although the BRFSS caregiver module is not administered in all US states annually, combining the surveys from 2020–2022 allowed for coverage of all 50 states [14–16].

### Measures

We obtained information on rural/urban county of residence status based on the BRFSS question, “Urban/Rural Status”, where response categories were either “Urban counties” or “Rural counties” [17]. Designation of urban or rural county status in this BRFSS question is based off the National Center for Health Statistics Urban-Rural Classification scheme where “Large Central Metro”, “Large fringe metro”, “Medium metro”, “Small metro”, and “Micropolitan” counties are considered “Urban counties” and “Noncore” counties are considered “Rural counties” [17,18]. Outcomes of interest were mental health and physical health within previous month which were categorized as follows: 0 poor PMHD or PPHD, 1–13 PMHD or PPHD, or 14 + PMHD or PPHD. These categorizations were taken directly from the BRFSS’s “Computed Mental Health Status” and “Computed Physical Health Status” variables [13,17]. Furthermore, prior literature has established PMHD and PPHD as reliable and valid self-reported mental and physical health indicators [19]. Caregivers of PWD were excluded if they were missing information on rural/urban status and mental and physical health within past month. However, caregivers of PWD with information on rural/urban status and mental and physical health within past month were not further excluded if they were missing data on the sociodemographic characteristics and caregiving factors described below.

Covariates in this study were chosen a priori based in accordance with the stress process model for caregivers of PWD [20]. These included sociodemographic characteristics (age, sex, race/ethnicity, household size, employment, education, income, health insurance, personal doctor) and caregiving factors (caregiving relationship, caregiving hours) from caregivers of a person living with dementia or other cognitive impairment disorder. The BRFSS survey questions used to collect this information and details on how variables were defined are included in S1 Table.

### Statistical analyses

We determined the distribution of sociodemographic characteristics and caregiving factors among rural and urban caregivers of PWD. Before conducting statistical modelling, we imputed missing values for sociodemographic and caregiving factors using a technique called fractional hot-deck imputation that accounts for survey weighting. Briefly, hot deck imputation takes an individual with a missing value on say variable A who is known as a recipient and replaces that individual’s missing variable A value with the observed variable A value from an individual known as a donor who has comparable values on observed variables B and C as the recipient [21]. With fractional hot deck imputation, rather than one donor’s observed value being used to replace the missing recipient value, several donors with comparable characteristics to the recipient will contribute to filling in the missing recipient value by a fraction of the original weight of the recipient [21]. With this method, we filled in missing data for race/ethnicity, household size, employment, income, education, health insurance, personal doctor, caregiving relationship, and caregiving hours as these variables were found to have missing values from our descriptive analyses (Table 1).

**Table 1:** Characteristics of caregivers of individuals with Alzheimer’s disease, dementia or other cognitive impairment disorder (N = 17,405)^1^.

Characteristic	Urban (N = 15,094)		Rural (N = 2311)	
	n	Survey Weighted % (95% CI)	n	Survey Weighted % (95% CI)
**Age**				
18-44	2480	26.4 (20.7, 32.2)	389	31.7 (14.8, 48.7)
45-64	7010	48.3 (42.9, 53.7)	1046	46.6 (32.2, 61.1)
65 and older	5604	25.3 (21.3, 29.3)	876	21.6 (14.8, 28.5)
**Sex**				
Male	5199	40.4 (35.0, 45.9)	720	35.0 (22.1, 47.8)
Female	9895	59.6 (54.1, 65.0)	1591	65.0 (52.2, 77.9)
**Race/Ethnicity**				
White only, Non-Hispanic	11810	72.4 (67.2, 77.6)	1948	85.9 (78.9, 92.8)
Black only, Non-Hispanic	1255	12.5 (8.0, 17.0)	134	4.7 (3.0, 6.4)
Other race only, Non-Hispanic	590	4.0 (1.5, 6.5)	87	1.3 (0.6, 2.0)
Multiracial, Non-Hispanic	368	2.1 (0.6, 3.5)	44	0.9 (0.4, 1.3)
Hispanic	746	6.9 (4.6, 9.2)	49	4.8 (0.0, 10.8)
Missing	325	2.2 (1.0, 3.4)	49	2.4 (0.2, 4.6)
**Household Size**				
1 person	2745	13.6 (9.4, 17.8)	453	12.7 (8.1, 17.4)
2-4 people	10691	72.1 (66.8, 77.3)	1633	74.0 (64.5, 83.4)
>4 people	1520	13.1 (9.1, 17.1)	213	12.8 (5.5, 20.1)
Missing	138	1.3 (0.0, 2.6)	12	0.5 (0.0, 1.0)
**Employment**				
Employed for wages	5678	43.8 (38.4, 49.2)	820	39.3 (25.6, 53.1)
Self-employed	1330	10.4 (6.2, 14.5)	235	6.0 (3.8, 8.2)
Out of work for 1 year or more	466	4.1 (2.0, 6.2)	60	2.3 (0.7, 3.8)
Out of work for < 1 year	343	1.7 (0.9, 2.4)	43	13.1 (0.0, 32.8)
Out of the work force (includes homemaker, a student, retired, unable to work)	7173	39.1 (34.1, 44.0)	1142	39.2 (26.7, 51.6)
Missing	104	1.0 (0.0, 2.3)	11	0.1 (0.0, 0.2)
**Income**				
<$15,000	731	3.4 (2.4, 4.4)	194	8.6 (3.7, 13.4)
$15,000- < $25,000	1349	9.6 (6.4, 12.8)	272	13.0 (4.5, 21.4)
$25,000- < $35,000	1539	9.6 (6.8, 12.5)	285	24.1 (5.9, 42.3)
$35,000- < $50,000	1852	12.5 (8.2, 16.8)	322	9.9 (6.2, 13.6)
$50,000 or more	7296	45.5 (40.1, 50.8)	896	33.7 (20.9, 46.6)
Missing	2327	19.4 (15.2, 23.6)	342	10.7 (7.2, 14.3)
**Education**				
Did not complete high school	558	4.9 (3.3, 6.6)	127	7.8 (4.7, 10.8)
High school gradate	3165	33.1 (27.3, 39.0)	641	38.4 (24.5, 52.4)
Some college or technical school	4598	32.8 (27.9, 37.6)	762	38.3 (22.3, 54.4)
College graduate	6737	29.0 (25.0, 33.1)	779	15.5 (10.3, 20.7)
Missing	36	0.1 (0.1, 0.2)	2	0.0 (0.0, 0.1)
**Health Insurance**				
Yes	14075	92.8 (90.7, 94.9)	2137	87.9 (81.1, 94.8)
No	663	3.9 (2.7, 5.2)	133	9.5 (3.2, 15.9)
Missing	356	3.3 (1.6, 4.9)	41	2.5 (0.2, 4.8)
**Personal Doctor**				
Yes, only one	9243	59.7 (54.5, 64.9)	1474	62.9 (46.9, 78.9)
More than one	4665	32.4 (27.3, 37.5)	627	30.3 (13.3, 47.2)
No	1111	7.6 (5.3, 10)	199	6.6 (3.9, 9.4)
Missing	75	0.3 (0.2, 0.4)	11	0.2 (0.0, 0.4)
**Caregiving Relationship**				
Child	861	6.0 (3.6, 8.3)	116	3.1 (1.8, 4.3)
Non-relative/Family Friend	2037	12.0 (8.7, 15.3)	369	9.1 (5.8, 12.4)
Other relative	2711	23.3 (17.7, 28.9)	432	20 (11.7, 28.3)
Parent/Parent in law	6765	43.5 (38.4, 48.6)	964	44.1 (29.9, 58.4)
Spouse/Live-In partner	2564	13.4 (10.1, 16.7)	415	22.3 (4.3, 40.3)
Missing	156	1.9 (0.0, 4.1)	15	1.4 (0.0, 3.5)
**Caregiving Hours**				
Up to 8 hours/week	6275	37.1 (32.4, 41.9)	985	32.1 (21.6, 42.6)
9 to 19 hours/week	2213	18.7 (13.5, 24.0)	299	22.1 (3.5, 40.6)
20 to 39 hours/week	1977	15.6 (11.4, 19.9)	301	16.9 (7.3, 26.6)
40 hours or more/week	3790	23.5 (18.9, 28.2)	607	26.0 (14.4, 37.6)
Missing	839	4.9 (3.1, 6.8)	119	2.9 (1.5, 4.3)
**Self-reported poor mental health days in past month**				
0 days	7760	51.6 (46.2, 56.9)	1224	56.4 (42.9, 70.0)
1-13 days	4457	27.6 (23.2, 32.1)	618	17.9 (11.9, 23.9)
14 + days	2877	20.8 (16.5, 25.1)	469	25.7 (14.6, 36.7)
**Physical Health in Past Month**				
0 days when physical health not good	9073	57.5 (52.1, 62.9)	1300	49.8 (35.1, 64.4)
1-13 days when physical health not good	3831	30.0 (24.7, 35.4)	606	22.5 (14.8, 30.2)
14 + days when physical health not good	2190	12.5 (9.9, 15.0)	405	27.7 (10.0, 45.5)

^1^Note that there are still missing values in Table 1 as it shows the proportion of missing responses across sociodemographic variables in the study before imputation of missing values on sociodemographic variables.

As both the PMHD and PPHD outcomes had three categories, we used multinomial logistic models with 0 days as the reference. Four separate survey-weighted multinomial logistic models were created for each outcome: (1) unadjusted model with rural/urban status only, (2) adjusted model with sociodemographic factors, (3) adjusted model with sociodemographic and caregiving factors, and (4) adjusted model with sociodemographic and caregiving factors with covariates from backwards selection. Using the unadjusted model for PMHD as an example, the multinomial model fits one model that provides separate parameter coefficients for the outcome of 1–13 PMHD vs 0 days and the outcome of 14 + PMHD vs 0 days but gives the same overall Chi-squared test p value for a predictor variable when comparing 1–13 PMHD vs 0 days and comparing 14 + PPHD vs 0 days.

The process of creating the models described in (4) above allowed for the examination of two-way interactions between rural/urban status and age, sex, race/ethnicity and the interplay between mental and physical health on outcomes. Covariates for the backward selection process in models described in (4) included sociodemographic and caregiving factors, physical/mental health (based on the outcome), and two-way interactions between rural/urban status and age, sex, and race/ethnicity. S2-S3 Tables show the selection results. Sociodemographic and caregiving factors were retained regardless of selection outcomes due to their relevance to physical/mental health, while interactions and physical/mental health covariates were retained only if included in the final selection model.

For sensitivity analyses, the four survey-weighted multinomial logistic models for each outcome were rerun using a dataset without imputed sociodemographic and caregiving factors. Models in (2) used data with 19.0% missing, while models in (3) and (4) used data with 23.1% missing. Results are shown in S4-S7 Tables. All analyses were performed using SAS V9.4 software with survey-weighting applied throughout. Statistical testing was conducted at α = 0.05.

### Ethics

The authors did not seek ethics approval and informed consent for this study as the BRFSS data that was used falls under the exemption status for IRB review at the authors’ institution. Additionally, the BRFSS has already undergone the CDC’s IRB and informed consent process before the survey was administered. Furthermore, the CDC only offers deidentified BRFSS surveys for public use, meaning investigators using these surveys are exempt from needing to seek additional informed consent or IRB review at their institution.

## Results

Our study was comprised of 17,405 caregivers of PWD (13.3% in rural areas (Table 1). The survey-weighted proportion of women was respectively 65.0% and 59.6% in rural and urban areas. A larger proportion of rural compared to urban caregivers were white (85.9% vs 72.4%), but the proportion 65 + years was similar by residence (rural: 21.6%, urban: 25.3%). Overall, 48.4% and 43.6% of rural and urban caregivers reported ≥1 PMHD in the previous month; among which, 25.7% (95% CI: 14.6%−36.7%) of rural and 20.8% (95% CI: 16.5%−25.1%) of urban caregivers reported 14 + PMHD. For physical health, 50.2% and 42.5% of rural and urban caregivers had ≥ 1 poor PPHD in the previous month with 27.7% (95% CI: (10.0%−45.5%) and 12.5% (95% CI: 9.9%−15%) of rural and urban caregivers respectively having 14 + PPHD.

Prior to adjustment for sociodemographic and caregiving factors, rural caregivers had 0.59 (95% CI: 0.34–1.05) times the odds (41% lower odds) of having 1–13 vs 0 PMHD and 1.13 (95% CI: 0.55–2.30) times the odds (13% higher odds) of having 14 + vs 0 PMHD compared to urban caregivers with these results not being statistically significant (Tables 2 and 3). For 1–13 vs 0 PMHD, associations between rural residence and mental health remained similar in all adjusted models with these results being statistically significant. In contrast, rural residence was associated with lower odds of 14 + vs 0 PMHD across all three adjusted models, though these results were not significant. In all three adjusted models comparing 1–13 vs 0 PMHD and 14 + vs 0 PMHD, income was the only sociodemographic factor significantly associated with PMHD. Of note, increasing income appeared to be strongly protective against PMHD. Caregiving hours displayed a significant association with PMHD for 1–13 vs 0 PMHD and 14 + vs 0 PMHD in all adjusted models. Sensitivity analyses using non-imputed data (S3 Table) indicated that rural residence was associated with lower odds of both 1–13 vs. 0 PMHD and 14 + vs. 0 PMHD in all adjusted models. However, these sensitivity analyses results should be interpreted with caution due to the high level of missing data.

**Table 2:** Associations between rural/urban residence, sociodemographic factors, and caregiving factors on mental health in previous month comparing outcome of 1-13 poor mental health days vs 0 days.

Covariate	Unadjusted Model^2^		Adjusted Model (sociodemographic factors)^3^		Adjusted Model (sociodemographic and caregiving factors)^4^		Adjusted Model (sociodemographic and caregiving factors) with covariates from backwards selection^5^	
	Odds Ratio	P-value	Odds Ratio	P-value	Odds Ratio	P-value^6^	Odds Ratio	P-value
	(1–13 days when mental health not good vs 0 days when mental health not good)		(1–13 days when mental health not good vs 0 days when mental health not good)		(1–13 days when mental health not good vs 0 days when mental health not good)		(1–13 days when mental health not good vs 0 days when mental health not good)	
**Rural/Urban status** **(ref: Urban)**		0.0361		0.0658		0.0956		0.1168
Rural^7^	0.59 (0.34, 1.05)		0.55 (0.31, 0.95)*		0.57 (0.33, 0.99)*		0.56 (0.33, 0.97)*	
**Age** **(ref: 18–44)**		–		0.0041		0.0108		0.1234
45-64	–		0.68 (0.36, 1.28)		0.7 (0.38, 1.29)		0.71 (0.39, 1.31)	
65 and older	–		0.45 (0.22, 0.93)		0.55 (0.26, 1.15)		0.62 (0.29, 1.29)	
**Sex** **(ref: Male)**		–		0.0369		0.0511		0.0561
Female	–		1.22 (0.79, 1.89)		1.33 (0.88, 1.99)		1.23 (0.81, 1.87)	
**Race/Ethnicity** **(ref: White only, Non-Hispanic)**		–		0.1261		0.076		0.0292
Black only, Non-Hispanic	–		1.21 (0.59, 2.50)		1.27 (0.64, 2.51)		1.11 (0.59, 2.09)	
Other race only, Non-Hispanic	–		0.41 (0.14, 1.24)		0.45 (0.18, 1.12)		0.40 (0.16, 1.00)	
Multiracial, Non-Hispanic	–		1.94 (0.36, 10.48)		1.93 (0.47, 7.98)		2.60 (0.58, 11.73)	
Hispanic	–		0.63 (0.26, 1.51)		0.73 (0.37, 1.47)		0.80 (0.41, 1.57)	
**Household Size** **(ref: 1 person)**		–		0.3249		0.5018		0.1138
2-4 people	–		1.11 (0.59, 2.07)		1.18 (0.61, 2.27)		1.17 (0.64, 2.16)	
>4 people	–		1.43 (0.62, 3.30)		1.48 (0.66, 3.32)		1.52 (0.74, 3.11)	
**Employment** **(ref: Employed for wages)**		–		0.084		0.0379		0.2592
Self-employed	–		0.55 (0.23, 1.30)		0.48 (0.20, 1.13)		0.52 (0.24, 1.17)	
Out of work for 1 year or more	–		0.87 (0.27, 2.86)		0.85 (0.27, 2.65)		0.75 (0.21, 2.67)	
Out of work for < 1 year	–		0.47 (0.17, 1.30)		0.48 (0.19, 1.25)		0.51 (0.20, 1.30)	
Out of the work force (includes homemaker, a student, retired, unable to work)	–		0.73 (0.44, 1.23)		0.73 (0.45, 1.20)		0.75 (0.46, 1.24)	
**Education** **(ref: College graduate)**		–		0.084		0.0497		0.1784
Did not complete high school	–		0.66 (0.35, 1.25)		0.61 (0.31, 1.18)		0.58 (0.29, 1.15)	
High school graduate	–		0.56 (0.31, 1.01)		0.57 (0.32, 1.01)		0.51 (0.28, 0.95)*	
Some college or technical school	–		0.92 (0.57, 1.48)		1.01 (0.65, 1.56)		0.93 (0.61, 1.41)	
**Income** **(ref: < $15,000)**		–		<.0001		0.0002		0.0086
$15,000- < $25,000	–		0.98 (0.42, 2.29)		0.96 (0.44, 2.11)		0.87 (0.41, 1.87)	
$25,000- < $35,000	–		0.83 (0.37, 1.86)		0.76 (0.35, 1.66)		0.77 (0.36, 1.66)	
$35,000- < $50,000	–		0.42 (0.19, 0.92)*		0.43 (0.20, 0.92)*		0.41 (0.18, 0.93)*	
$50,000 or more	–		0.54 (0.28, 1.06)		0.53 (0.28, 1.02)		0.55 (0.28, 1.07)	
**Health Insurance** **(ref: No)**		–		0.6227		0.4524		0.2566
Yes	–		1.19 (0.52, 2.70)		1.24 (0.55, 2.82)		1.04 (0.49, 2.19)	
**Personal Doctor** **(ref: No)**		–		0.1614		0.0795		0.0898
Yes, only one	–		1.37 (0.66, 2.85)		1.31 (0.64, 2.69)		1.42 (0.64, 3.12)	
More than one	–		0.95 (0.43, 2.11)		0.87 (0.40, 1.88)		0.82 (0.34, 1.93)	
**Caregiving Relationship** **(ref: Non-relative/Family Friend)**		–		–		0.1898		0.2036
Child	–		–		1.78 (0.69, 4.58)		1.78 (0.70, 4.54)	
Other relative	–		–		1.24 (0.54, 2.86)		1.28 (0.53, 3.12)	
Parent/Parent in law	–		–		1.36 (0.68, 2.71)		1.36 (0.66, 2.81)	
Spouse/Live-In partner	–		–		0.69 (0.30, 1.58)		0.77 (0.33, 1.80)	
**Caregiving Hours** **(ref: Up to 8 hours/week)**		–		–		0.0119		0.0112
9 to 19 hours/week	–		–		1.51 (0.85, 2.65)		1.37 (0.76, 2.46)	
20 to 39 hours/week	–		–		0.46 (0.22, 0.94)*		0.45 (0.22, 0.93)*	
40 hours or more/week	–		–		1.10 (0.63, 1.95)		1.06 (0.59, 1.92)	
**Physical Health** **(ref: 0 days)**		–		–		–		<.0001
1-13 days	–		–		–		3.13 (1.94, 5.03)	
14 + days	–		–		–		1.65 (0.92, 2.97)	

^2^Model includes rural/urban status only.

^3^Model covariates include rural/urban status and sociodemographic factors (age, sex, race/ethnicity, household size, employment, education, income, health insurance, personal doctor).

^4^Model covariates include rural/urban status, sociodemographic factors (age, sex, race/ethnicity, household size, employment, education, income, health insurance, personal doctor), and caregiving factors (caregiving relationship, caregiving hours).

^5^Model covariates include rural/urban status, sociodemographic factors (age, sex, race/ethnicity, household size, employment, education, income, health insurance, personal doctor), caregiving factors (caregiving relationship, caregiving hours), and physical health.

^6^For a multinomial logistic model, the overall Chi-squared test p value for a variable (i.e., race, sex education) will be the same for a variable comparing 1–13 poor mental health days vs 0 days (Table 2) and comparing 14 + poor mental health days vs 0 days (Table 3).

^7^An asterisk (*) indicates that a category is statistically significant (p-value<0.05) from the reference category in terms of the outcome.

**Table 3:** Associations between rural/urban residence, sociodemographic factors, and caregiving factors on mental health in previous month comparing outcome of 14 + poor mental health days vs 0 days.

Covariate	Unadjusted Model^8^		Adjusted Model (sociodemographic factors)^9^		Adjusted Model (sociodemographic and caregiving factors)^10^		Adjusted Model (sociodemographic and caregiving factors) with covariates from backwards selection^11^	
	Odds Ratio	P-value	Odds Ratio	P-value	Odds Ratio	P-value^12^	Odds Ratio	P-value
	(14 + days when mental health not good vs 0 days when mental health not good)		(14 + days when mental health not good vs 0 days when mental health not good)		(14 + days when mental health not good vs 0 days when mental health not good)		(14 + days when mental health not good vs 0 days when mental health not good)	
**Rural/Urban status** **(ref: Urban)**		0.0361		0.0658		0.0956		0.1168
Rural^13^	1.13 (0.55, 2.30)		0.89 (0.45, 1.78)		0.88 (0.45, 1.71)		0.66 (0.29, 1.54)	
**Age** **(ref: 18–44)**		–		0.0041		0.0108		0.1234
45-64	–		0.59 (0.31, 1.11)		0.67 (0.35, 1.29)		0.61 (0.31, 1.19)	
65 and older	–		0.26 (0.13, 0.52)*		0.27 (0.13, 0.56)*		0.36 (0.17, 0.76)*	
**Sex** **(ref: Male)**		–		0.0369		0.0511		0.0561
Female	–		1.85 (1.16, 2.96)		1.71 (1.10, 2.66)		1.80 (1.11, 2.91)	
**Race/Ethnicity** **(ref: White only, Non-Hispanic)**		–		0.1261		0.076		0.0292
Black only, Non-Hispanic	–		0.75 (0.35, 1.63)		0.64 (0.31, 1.33)		0.54 (0.25, 1.15)	
Other race only, Non-Hispanic	–		1.55 (0.44, 5.49)		1.33 (0.38, 4.71)		1.38 (0.38, 5.05)	
Multiracial, Non-Hispanic	–		1.6 (0.41, 6.28)		1.32 (0.35, 4.97)		1.46 (0.45, 4.76)	
Hispanic	–		0.56 (0.27, 1.15)		0.5 (0.22, 1.12)		0.47 (0.21, 1.03)	
**Household Size** **(ref: 1 person)**		–		0.3249		0.5018		0.1138
2-4 people	–		1.30 (0.74, 2.30)		1.30 (0.71, 2.39)		1.60 (0.86, 2.95)	
>4 people	–		2.51 (1.08, 5.84)		2.28 (0.94, 5.52)		3.30 (1.40, 7.77)	
**Employment** **(ref: Employed for wages)**		–		0.084		0.0379		0.2592
Self-employed	–		0.84 (0.39, 1.78)		0.79 (0.38, 1.64)		0.80 (0.39, 1.62)	
Out of work for 1 year or more	–		1.78 (0.54, 5.79)		1.72 (0.53, 5.56)		1.32 (0.38, 4.57)	
Out of work for < 1 year	–		0.36 (0.09, 1.45)		0.38 (0.10, 1.41)		0.23 (0.04, 1.22)	
Out of the work force (includes homemaker, a student, retired, unable to work)	–		1.80 (1.00, 3.26)		1.74 (0.97, 3.14)		1.31 (0.74, 2.34)	
**Education** **(ref: College graduate)**		–		0.084		0.0497		0.1784
Did not complete high school	–		1.98 (0.91, 4.28)		1.92 (0.90, 4.11)		1.24 (0.58, 2.65)	
High school graduate	–		0.80 (0.43, 1.46)		0.72 (0.40, 1.30)		0.63 (0.34, 1.15)	
Some college or technical school	–		1.38 (0.73, 2.59)		1.28 (0.70, 2.36)		1.18 (0.63, 2.19)	
**Income** **(ref: < $15,000)**		–		<.0001		0.0002		0.0086
$15,000- < $25,000	–		0.90 (0.41, 1.98)		0.89 (0.42, 1.88)		1.01 (0.47, 2.17)	
$25,000- < $35,000	–		0.68 (0.30, 1.54)		0.71 (0.32, 1.59)		0.79 (0.33, 1.85)	
$35,000- < $50,000	–		0.32 (0.15, 0.71)*		0.32 (0.15, 0.69)*		0.43 (0.20, 0.94)*	
$50,000 or more	–		0.23 (0.12, 0.44)*		0.23 (0.12, 0.44)*		0.31 (0.16, 0.59)*	
**Health Insurance** **(ref: No)**		–		0.6227		0.4524		0.2566
Yes	–		0.86 (0.42, 1.76)		0.81 (0.40, 1.64)		0.62 (0.29, 1.32)	
**Personal Doctor** **(ref: No)**		–		0.1614		0.0795		0.0898
Yes, only one	–		0.78 (0.35, 1.74)		0.81 (0.37, 1.78)		0.79 (0.32, 1.96)	
More than one	–		1.04 (0.44, 2.45)		1.09 (0.47, 2.52)		0.74 (0.29, 1.90)	
**Caregiving Relationship** **(ref: Non-relative/Family Friend)**		–		–		0.1898		0.2036
Child	–		–		0.73 (0.27, 1.99)		0.74 (0.28, 1.96)	
Other relative	–		–		0.91 (0.42, 1.97)		1.15 (0.52, 2.54)	
Parent/Parent in law	–		–		0.63 (0.30, 1.32)		0.63 (0.30, 1.36)	
Spouse/Live-In partner	–		–		0.74 (0.31, 1.77)		0.75 (0.30, 1.85)	
**Caregiving Hours** **(ref: Up to 8 hours/week)**		–		–		0.0119		0.0112
9 to 19 hours/week	–		–		1.30 (0.72, 2.37)		1.27 (0.67, 2.42)	
20 to 39 hours/week	–		–		1.89 (0.87, 4.13)		2.25 (1.04, 4.90)	
40 hours or more/week	–		–		1.36 (0.78, 2.37)		1.20 (0.66, 2.18)	
**Physical Health** **(ref: 0 days)**		–		–		–		<.0001
1-13 days	–		–		–		2.78 (1.43, 5.39)*	
14 + days	–		–		–		10.75 (6.19, 18.69)*	
**Rural/Urban status** **(ref: Urban)**		0.0361		0.0658		0.0956		0.1168
Rural^8^	1.13 (0.55, 2.30)		0.89 (0.45, 1.78)		0.88 (0.45, 1.71)		0.66 (0.29, 1.54)	
**Age** **(ref: 18–44)**		–		0.0041		0.0108		0.1234
45-64	–		0.59 (0.31, 1.11)		0.67 (0.35, 1.29)		0.61 (0.31, 1.19)	
65 and older	–		0.26 (0.13, 0.52)*		0.27 (0.13, 0.56)*		0.36 (0.17, 0.76)*	
**Sex** **(ref: Male)**		–		0.0369		0.0511		0.0561
Female	–		1.85 (1.16, 2.96)		1.71 (1.10, 2.66)		1.80 (1.11, 2.91)	
**Race/Ethnicity** **(ref: White only, Non-Hispanic)**		–		0.1261		0.076		0.0292
Black only, Non-Hispanic	–		0.75 (0.35, 1.63)		0.64 (0.31, 1.33)		0.54 (0.25, 1.15)	
Other race only, Non-Hispanic	–		1.55 (0.44, 5.49)		1.33 (0.38, 4.71)		1.38 (0.38, 5.05)	
Multiracial, Non-Hispanic	–		1.6 (0.41, 6.28)		1.32 (0.35, 4.97)		1.46 (0.45, 4.76)	
Hispanic	–		0.56 (0.27, 1.15)		0.5 (0.22, 1.12)		0.47 (0.21, 1.03)	
**Household Size** **(ref: 1 person)**		–		0.3249		0.5018		0.1138
2-4 people	–		1.30 (0.74, 2.30)		1.30 (0.71, 2.39)		1.60 (0.86, 2.95)	
>4 people	–		2.51 (1.08, 5.84)		2.28 (0.94, 5.52)		3.30 (1.40, 7.77)	
**Employment** **(ref: Employed for wages)**		–		0.084		0.0379		0.2592
Self-employed	–		0.84 (0.39, 1.78)		0.79 (0.38, 1.64)		0.80 (0.39, 1.62)	
Out of work for 1 year or more	–		1.78 (0.54, 5.79)		1.72 (0.53, 5.56)		1.32 (0.38, 4.57)	
Out of work for < 1 year	–		0.36 (0.09, 1.45)		0.38 (0.10, 1.41)		0.23 (0.04, 1.22)	
Out of the work force (includes homemaker, a student, retired, unable to work)	–		1.80 (1.00, 3.26)		1.74 (0.97, 3.14)		1.31 (0.74, 2.34)	
**Education** **(ref: College graduate)**		–		0.084		0.0497		0.1784
Did not complete high school	–		1.98 (0.91, 4.28)		1.92 (0.90, 4.11)		1.24 (0.58, 2.65)	
High school graduate	–		0.80 (0.43, 1.46)		0.72 (0.40, 1.30)		0.63 (0.34, 1.15)	
Some college or technical school	–		1.38 (0.73, 2.59)		1.28 (0.70, 2.36)		1.18 (0.63, 2.19)	
**Income** **(ref: < $15,000)**		–		<.0001		0.0002		0.0086
$15,000- < $25,000	–		0.90 (0.41, 1.98)		0.89 (0.42, 1.88)		1.01 (0.47, 2.17)	
$25,000- < $35,000	–		0.68 (0.30, 1.54)		0.71 (0.32, 1.59)		0.79 (0.33, 1.85)	
$35,000- < $50,000	–		0.32 (0.15, 0.71)*		0.32 (0.15, 0.69)*		0.43 (0.20, 0.94)*	
$50,000 or more	–		0.23 (0.12, 0.44)*		0.23 (0.12, 0.44)*		0.31 (0.16, 0.59)*	
**Health Insurance** **(ref: No)**		–		0.6227		0.4524		0.2566
Yes	–		0.86 (0.42, 1.76)		0.81 (0.40, 1.64)		0.62 (0.29, 1.32)	
**Personal Doctor** **(ref: No)**		–		0.1614		0.0795		0.0898
Yes, only one	–		0.78 (0.35, 1.74)		0.81 (0.37, 1.78)		0.79 (0.32, 1.96)	
More than one	–		1.04 (0.44, 2.45)		1.09 (0.47, 2.52)		0.74 (0.29, 1.90)	
**Caregiving Relationship** **(ref: Non-relative/Family Friend)**		–		–		0.1898		0.2036
Child	–		–		0.73 (0.27, 1.99)		0.74 (0.28, 1.96)	
Other relative	–		–		0.91 (0.42, 1.97)		1.15 (0.52, 2.54)	
Parent/Parent in law	–		–		0.63 (0.30, 1.32)		0.63 (0.30, 1.36)	
Spouse/Live-In partner	–		–		0.74 (0.31, 1.77)		0.75 (0.30, 1.85)	
**Caregiving Hours** **(ref: Up to 8 hours/week)**		–		–		0.0119		0.0112
9 to 19 hours/week	–		–		1.30 (0.72, 2.37)		1.27 (0.67, 2.42)	
20 to 39 hours/week	–		–		1.89 (0.87, 4.13)		2.25 (1.04, 4.90)	
40 hours or more/week	–		–		1.36 (0.78, 2.37)		1.20 (0.66, 2.18)	
**Physical Health** **(ref: 0 days)**		–		–		–		<.0001
1-13 days	–		–		–		2.78 (1.43, 5.39)*	
14 + days	–		–		–		10.75 (6.19, 18.69)*	

^8^Model includes rural/urban status only.

^9^Model covariates include rural/urban status and sociodemographic factors (age, sex, race/ethnicity, household size, employment, education, income, health insurance, personal doctor).

^10^Model covariates include rural/urban status and sociodemographic factors (age, sex, race/ethnicity, household size, employment, education, income, health insurance, personal doctor).and caregiving factors (caregiving relationship, caregiving hours).

^11^Model covariates include rural/urban status, sociodemographic factors (age, sex, race/ethnicity, household size, employment, education, income, health insurance, personal doctor), caregiving factors (caregiving relationship, caregiving hours), and physical health.

^12^For a multinomial logistic model, the overall Chi-squared test p value for a variable (i.e., race, sex education) will be the same for a variable comparing 1–13 poor mental health days vs 0 days (Table 2) and comparing 14 + poor mental health days vs 0 days (Table 3).

^13^An asterisk (*) indicates that a category is statistically significant (p-value<0.05) from the reference category in terms of the outcome.

Rural caregivers had 0.86 (95% CI: 0.53–1.41) times the odds (14% lower odds) of 1–13 vs 0 PPHD and 2.57 (95% CI: 1.00–6.63) times the odds (157% higher odds) of 14 + vs 0 PPHD compared to urban caregivers with these findings not being statistically significant (Tables 4 and 5). For 1–13 vs 0 PPHD days, the association between rural residence and physical health was attenuated in all adjusted models and remained statistically not significant. Of note, rural caregivers had significantly higher odds of 14 + vs. 0 PPHD days compared to urban caregivers, with adjusted models showing a magnitude of association similar to the unadjusted model. Sociodemographic factors significantly associated with PPHD for all 1–13 vs 0 and 14 + vs 0 PPHD adjusted models included age, race/ethnicity, employment, income, and having a personal doctor. Caregiving relationship was significantly associated with PPHD in all 1–13 vs 0 PPHD and 14 + vs 0 PPHD adjusted models. For the sake of brevity, readers can refer to the table for the directionality of each category of the different variables significantly associated with the outcome across the adjusted 1–13 vs 0 days and 14 + vs 0 days models. Sensitivity analyses for the physical health outcome using non-imputed data indicated that rural residence was linked to lower odds of 1–13 vs 0 PPHD but higher odds of 14 + vs 0 PPHD. Similar to the mental health outcome models, these results should be interpreted cautiously due to the high level of missing data.

**Table 4:** Associations between rural/urban residence, sociodemographic factors, and caregiving factors on physical health in previous month comparing outcome of 1-13 poor physical health days vs 0 days.

Covariate	Unadjusted Model^14^		Adjusted Model (sociodemographic factors)^15^		Adjusted Model (sociodemographic and caregiving factors)^16^		Adjusted Model (sociodemographic and caregiving factors) with covariates from backwards selection^17^	
	Odds Ratio	P-value	Odds Ratio	P-value	Odds Ratio	P-value^18^	Odds Ratio	P-value
	(1–13 days when physical health not good vs 0 days when physical health not good)		(1–13 days when physical health not good vs 0 days when physical health not good)		(1–13 days when physical health not good vs 0 days when physical health not good)		(1–13 days when physical health not good vs 0 days when physical health not good)	
**Rural/Urban status** **(ref: Urban)**		0.0734		0.0288		0.0327		0.0326
Rural^19^	0.86 (0.53, 1.41)		0.87 (0.50, 1.54)		0.90 (0.50, 1.63)		1.00 (0.55, 1.82)	
**Age** **(ref: 18–44)**		–		<.0001		0.0031		0.0031
45-64	–		0.92 (0.50, 1.68)		0.98 (0.52, 1.86)		1.09 (0.59, 2.01)	
65 and older	–		0.44 (0.22, 0.88)*		0.56 (0.28, 1.14)		0.66 (0.33, 1.31)	
**Sex** **(ref: Male)**		–		0.4331		0.2921		0.3079
Female	–		1.34 (0.85, 2.12)		1.41 (0.90, 2.21)		1.31 (0.82, 2.09)	
**Race/Ethnicity** **(ref: White only, Non-Hispanic)**		–		0.0131		0.017		0.0107
Black only, Non-Hispanic	–		1.58 (0.74, 3.40)		1.62 (0.81, 3.24)		1.63 (0.83, 3.20)	
Other race only, Non-Hispanic	–		1.50 (0.46, 4.92)		1.35 (0.49, 3.71)		1.50 (0.59, 3.79)	
Multiracial, Non-Hispanic	–		0.25 (0.08, 0.78)*		0.22 (0.06, 0.82)*		0.17 (0.04, 0.82)*	
Hispanic	–		0.52 (0.25, 1.09)		0.54 (0.26, 1.11)		0.61 (0.32, 1.19)	
**Household Size** **(ref: 1 person)**		–		0.7238		0.6718		0.2754
2-4 people	–		0.95 (0.48, 1.87)		1.01 (0.51, 2.02)		0.94 (0.49, 1.78)	
>4 people	–		0.87 (0.34, 2.25)		0.91 (0.36, 2.30)		0.77 (0.32, 1.83)	
**Employment** **(ref: Employed for wages)**		–		0.0001		<.0001		0.0008
Self-employed	–		0.64 (0.28, 1.51)		0.56 (0.24, 1.32)		0.65 (0.29, 1.45)	
Out of work for 1 year or more	–		1.43 (0.50, 4.11)		1.27 (0.48, 3.39)		1.30 (0.41, 4.09)	
Out of work for < 1 year	–		0.56 (0.26, 1.24)		0.56 (0.26, 1.22)		0.64 (0.31, 1.3)	
Out of the work force (includes homemaker, a student, retired, unable to work)	–		0.87 (0.52, 1.44)		0.87 (0.54, 1.40)		0.89 (0.56, 1.44)	
**Education** **(ref: College graduate)**		–		0.0023		0.004		0.0051
Did not complete high school	–		1.33 (0.65, 2.69)		1.24 (0.59, 2.62)		1.37 (0.67, 2.83)	
High school graduate	–		1.56 (0.88, 2.76)		1.47 (0.87, 2.50)		1.68 (0.96, 2.93)	
Some college or technical school	–		1.37 (0.79, 2.38)		1.40 (0.85, 2.33)		1.38 (0.83, 2.27)	
**Income** **(ref: < $15,000)**		–		0.0002		0.0003		0.0621
$15,000- < $25,000	–		1.66 (0.71, 3.89)		1.64 (0.74, 3.62)		1.71 (0.75, 3.87)	
$25,000- < $35,000	–		0.89 (0.38, 2.08)		0.91 (0.41, 2.02)		0.95 (0.42, 2.13)	
$35,000- < $50,000	–		0.94 (0.40, 2.22)		0.99 (0.44, 2.26)		1.22 (0.51, 2.91)	
$50,000 or more	–		0.81 (0.39, 1.69)		0.83 (0.41, 1.68)		1.00 (0.47, 2.11)	
**Health Insurance** **(ref: No)**		–		0.1187		0.0963		0.0611
Yes	–		2.07 (0.88, 4.90)		2.09 (0.94, 4.66)		2.10 (1.00, 4.43)	
**Personal Doctor** **(ref: No)**		–		<.0001		<.0001		<.0001
Yes, only one	–		0.84 (0.40, 1.76)		0.76 (0.37, 1.57)		0.73 (0.33, 1.60)	
More than one	–		1.16 (0.52, 2.59)		1.06 (0.48, 2.33)		1.13 (0.48, 2.70)	
**Caregiving Relationship** **(ref: Non-relative/Family Friend)**		–		–		0.0421		0.0115
Child	–		–		0.82 (0.32, 2.09)		0.71 (0.28, 1.81)	
Other relative	–		–		0.77 (0.36, 1.63)		0.71 (0.32, 1.59)	
Parent/Parent in law	–		–		0.83 (0.43, 1.61)		0.79 (0.39, 1.59)	
Spouse/Live-In partner	–		–		0.41 (0.17, 0.95)*		0.42 (0.18, 1.01)	
**Caregiving Hours** **(ref: Up to 8 hours/week)**		–		–		0.1148		0.0172
9 to 19 hours/week	–		–		1.78 (1.00, 3.15)		1.66 (0.92, 3.01)	
20 to 39 hours/week	–		–		0.85 (0.35, 2.06)		0.92 (0.38, 2.22)	
40 hours or more/week	–		–		1.31 (0.78, 2.21)		1.33 (0.77, 2.31)	
**Mental Health** **(ref: 0 days)**		–		–		–		<.0001
1-13 days	–		–		–		3.14 (1.92, 5.14)*	
14 + days	–		–		–		2.48 (1.25, 4.95)*	

^14^Model includes rural/urban status only.

^15^Model covariates include rural/urban status and sociodemographic factors (age, sex, race/ethnicity, household size, employment, education, income, health insurance, personal doctor).

^16^Model covariates include rural/urban status, sociodemographic factors (age, sex, race/ethnicity, household size, employment, education, income, health insurance, personal doctor), and caregiving factors (caregiving relationship, caregiving hours).

^17^Model covariates include rural/urban status, sociodemographic factors (age, sex, race/ethnicity, household size, employment, education, income, health insurance, personal doctor), caregiving factors (caregiving relationship, caregiving hours), and mental health.

^18^For a multinomial logistic model, the overall Chi-squared test p value for a variable (i.e., race, sex education) will be the same for a variable comparing 1–13 poor physical health days vs 0 days (Table 2) and comparing 14 + poor physical health days vs 0 days (Table 5).

^19^An asterisk (*) indicates that a category is statistically significant (p-value<0.05) from the reference category in terms of the outcome.

**Table 5:** Associations between rural/urban residence, sociodemographic factors, and caregiving factors on physical health in previous month comparing 14 + poor physical health days vs 0 days.

Covariate	Unadjusted Model^20^		Adjusted Model (sociodemographic factors)^21^		Adjusted Model (sociodemographic and caregiving factors)^22^		Adjusted Model (sociodemographic and caregiving factors) with covariates from backwards selection^23^	
	Odds Ratio	P-value	Odds Ratio	P-value	Odds Ratio	P-value^24^	Odds Ratio	P-value
	(14 + days when physical health not good vs 0 days when physical health not good)		(14 + days when physical health not good vs 0 days when physical health not good)		(14 + days when physical health not good vs 0 days when physical health not good)		(14 + days when physical health not good vs 0 days when physical health not good)	
**Rural/Urban status** **(ref: Urban)**		0.0734		0.0288		0.0327		0.0326
Rural^25^	2.57 (1.00, 6.63)		2.02 (1.03, 3.95)*		2.01 (1.02, 3.95)*		2.26 (1.08, 4.76)*	
**Age** **(ref: 18–44)**		–		<.0001		0.0031		0.0031
45-64	–		1.79 (1.03, 3.11)*		1.48 (0.87, 2.53)		1.88 (1.10, 3.22)*	
65 and older	–		0.51 (0.22, 1.19)		0.42 (0.17, 1.08)		0.66 (0.24, 1.79)	
**Sex** **(ref: Male)**		–		0.4331		0.2921		0.3079
Female	–		1.04 (0.68, 1.60)		1.04 (0.68, 1.58)		0.90 (0.58, 1.39)	
**Race/Ethnicity** **(ref: White only, Non-Hispanic)**		–		0.0131		0.017		0.0107
Black only, Non-Hispanic	–		1.11 (0.59, 2.09)		1.25 (0.68, 2.30)		1.53 (0.84, 2.82)	
Other race only, Non-Hispanic	–		0.64 (0.26, 1.56)		0.6 (0.24, 1.50)		0.42 (0.13, 1.37)	
Multiracial, Non-Hispanic	–		0.86 (0.28, 2.63)		0.95 (0.30, 3.01)		0.78 (0.25, 2.37)	
Hispanic	–		1.63 (0.62, 4.33)		1.55 (0.58, 4.17)		2.03 (0.74, 5.59)	
**Household Size** **(ref: 1 person)**		–		0.7238		0.6718		0.2754
2-4 people	–		0.80 (0.44, 1.46)		0.72 (0.39, 1.35)		0.65 (0.35, 1.20)	
>4 people	–		0.50 (0.19, 1.32)		0.53 (0.21, 1.34)		0.36 (0.14, 0.91)*	
**Employment** **(ref: Employed for wages)**		–		0.0001		<.0001		0.0008
Self-employed	–		1.25 (0.48, 3.28)		1.19 (0.47, 3.00)		1.17 (0.45, 3.07)	
Out of work for 1 year or more	–		3.12 (1.03, 9.44)*		2.72 (0.92, 8.00)		2.29 (0.83, 6.36)	
Out of work for < 1 year	–		3.54 (1.09, 11.47)*		3.17 (1.00, 9.98)		4.61 (1.43, 14.89)*	
Out of the work force (includes homemaker, a student, retired, unable to work)	–		3.22 (1.83, 5.67)*		3.34 (1.91, 5.85)*		2.79 (1.55, 5.01)*	
**Education** **(ref: College graduate)**		–		0.0023		0.004		0.0051
Did not complete high school	–		4.21 (1.98, 8.95)*		3.78 (1.83, 7.82)*		3.32 (1.64, 6.69)*	
High school graduate	–		1.37 (0.74, 2.53)		1.36 (0.74, 2.51)		1.53 (0.79, 2.96)	
Some college or technical school	–		1.21 (0.68, 2.14)		1.19 (0.68, 2.08)		1.17 (0.65, 2.11)	
**Income** **(ref: < $15,000)**		–		0.0002		0.0003		0.0621
$15,000- < $25,000	–		0.81 (0.40, 1.65)		0.73 (0.36, 1.48)		0.73 (0.34, 1.58)	
$25,000- < $35,000	–		0.82 (0.36, 1.84)		0.80 (0.36, 1.79)		0.87 (0.38, 2.03)	
$35,000- < $50,000	–		0.29 (0.13, 0.61)*		0.28 (0.13, 0.61)*		0.38 (0.17, 0.83)*	
$50,000 or more	–		0.26 (0.14, 0.51)*		0.26 (0.14, 0.51)*		0.39 (0.20, 0.79)*	
**Health Insurance** **(ref: No)**		–		0.1187		0.0963		0.0611
Yes	–		2.70 (0.88, 8.31)		2.64 (0.87, 7.96)		2.87 (1.00, 8.24)	
**Personal Doctor** **(ref: No)**		–		<.0001		<.0001		<.0001
Yes, only one	–		0.94 (0.48, 1.86)		0.96 (0.50, 1.86)		1.06 (0.51, 2.24)	
More than one	–		3.12 (1.51, 6.44)*		3.07 (1.51, 6.25)*		3.40 (1.56, 7.40)*	
**Caregiving Relationship** **(ref: Non-relative/Family Friend)**		–		–		0.0421		0.0115
Child	–		–		1.01 (0.41, 2.47)		1.33 (0.56, 3.18)	
Other relative	–		–		0.43 (0.22, 0.85)*		0.46 (0.23, 0.93)*	
Parent/Parent in law	–		–		0.86 (0.45, 1.63)		1.14 (0.57, 2.26)	
Spouse/Live-In partner	–		–		0.82 (0.38, 1.73)		0.98 (0.45, 2.16)	
**Caregiving Hours** **(ref: Up to 8 hours/week)**		–		–		0.1148		0.0172
9 to 19 hours/week	–		–		1.15 (0.60, 2.21)		1.20 (0.58, 2.47)	
20 to 39 hours/week	–		–		0.67 (0.39, 1.15)		0.45 (0.25, 0.81)*	
40 hours or more/week	–		–		1.33 (0.80, 2.21)		1.17 (0.69, 1.99)	
**Mental Health** **(ref: 0 days)**		–		–		–		<.0001
1-13 days	–		–		–		1.66 (0.96, 2.88)	
14 + days	–		–		–		10.21 (6.01, 17.36)*	

^20^Model includes rural/urban status only.

^21^Model covariates include rural/urban status and sociodemographic factors (age, sex, race/ethnicity, household size, employment, education, income, health insurance, personal doctor).

^22^Model covariates include rural/urban status, sociodemographic factors (age, sex, race, education, household size, income, health insurance, personal doctor), and caregiving factors (caregiving relationship, caregiving hours).

^23^Model covariates include rural/urban status, sociodemographic factors (age, sex, race, education, household size, income, health insurance, personal doctor), caregiving factors (caregiving relationship, caregiving hours), and mental health.

^24^For a multinomial logistic model, the overall Chi-squared test p value for a variable (i.e., race, sex education) will be the same for a variable comparing 1–13 poor mental health days vs 0 days (Table 2) and comparing 14 + poor mental health days vs 0 days (Table 3).

^25^An asterisk (*) indicates that a category is statistically significant (p-value < 0.05) from the reference category in terms of the outcome.

## Discussion

Our study examined the relationship between rural/urban residence and poor mental and physical health in a nationally representative US sample of caregivers of PWD. Although rural caregivers of PWD were less likely to experience 1–13 PMHD compared to urban caregivers, they faced comparable levels of enduring frequent days of mental health challenges (14 + PMHD). Of note, over 1 in 4 rural caregivers and 1 in 5 urban caregivers reported 14 + PMHD in the previous month. Our findings suggest that, despite varying environmental factors, the mental health impacts of dementia caregiving are lasting across different residential settings. The similar mental health outcomes of rural and urban caregivers in this study align with prior research assessing differences in mental health between US rural and urban caregivers of PWD [9,10]. In a study of 212 caregivers of PWD enrolled in South Carolina’s Alzheimer’s Disease Registry in 2010, rural caregivers were 24% less likely to experience caregiver distress than caregivers in large urban areas. However, there was no significant difference in caregiver stress or burden between the two groups [9]. In a convenience-based sample of caregivers of PWD who were part of US Alzheimer and dementia support groups, researchers found that whether or not caregivers lived in rural or urban areas did not significantly impact their levels of caregiver grief, well-being, burden, quality of life, self-efficacy/mastery, or social network quality [10]. Building and extending this line of research, our study sheds additional light on proving estimated frequency of poor mental health based on a representative contemporary sample of US caregivers of PWD. Additionally, our findings are consistent with existing national studies that examined the general caregiver population across all illness contexts. These studies show that rural and urban caregivers were significantly more likely than their non-caregiver counterparts to report poor mental health [22,23]. Taken together, these findings indicate concerted efforts to improve long-term mental health support across rural and urban caregivers are warranted.

The findings of our study show that rural caregivers of PWD are vulnerable to more days of physical health challenges compared to their urban counterparts. This may be due to a combination of structural and environmental factors. First, rural caregivers are more likely to provide high-intensity caregiving. According to a national 2020 survey conducted by the American Association of Retired Persons, 34% of rural caregivers provide care to multiple family members compared to 23% of urban caregivers [24]. Rural caregivers of PWD also provide more hours of care per week than urban caregivers [25]. These increased caregiving demands may result in greater physical strain and fatigue over extended periods. Moreover, rural caregivers often face significant barriers when accessing health care services for PWD, due to geographic isolation, lack of transportation options, fewer healthcare facilities, and limited respite care services [26]. These challenges are compounded by the difficulty in finding financially affordable services, as reported by 32% of rural caregivers compared to 25% of urban caregivers [26]. Consequently, rural caregivers of PWD often shoulder the demanding responsibilities of caregiving on their own. This added strain can have a negative and lasting impact on their physical health.

Our findings highlight the importance of targeting programs and services to enhance the mental health of both rural and urban caregivers of PWD [27]. Given the different characteristics of rural and urban caregiving environments, improving the mental health of caregivers of PWD in these diverse settings require a multifaceted approach that recognizes the unique challenges they face. In rural areas, where there may be isolation and limited resources that can contribute to mental health struggles, programs should prioritize community engagement and outreach [28]. This could involve establishing local support networks, organizing informational sessions, and providing stress management workshops [29]. Additionally, telehealth services can bridge the gap by offering remote access to mental health professionals, ensuring timely and consistent support [30,31]. In urban settings, caregivers may experience heightened stress levels due to the fast-paced lifestyle, increased responsibilities, and limited access to support networks [32]. Initiatives should focus on creating accessible green space and convenient mental health resources, such as online counseling services or support groups that accommodate urban caregivers’ busy schedules [31,33]. Moreover, policymakers should advocate for flexible workplace policies to allow caregivers to better manage their personal and professional responsibilities. Flexible work arrangements, including remote work options and adjustable hours, can reduce stress for caregivers of PWD navigating demanding schedules. Last, urban multicomponent psychosocial interventions tailored toward the needs of urban caregivers of PWD may also reap lasting benefits [34].

The current study also identifies that rural caregivers of PWD experienced poor physical health. In some rural settings where geographical isolation and limited resources may impede physical health, interventions should prioritize community-based activities like group exercises or gardening initiatives to encourage both physical activity and social connections [35]. Additionally, given that rural caregivers are vulnerable to prolonged physical health challenges, advocating for policy changes to enhance healthcare accessibility, including increased respite care options and the implementation of telehealth programs, is imperative [36,37]. Although the interactions between rural/urban residence and sociodemographic characteristics (i.e., age, sex, and race/ethnicity) were not significant in the current study, additional research is still warranted to understand how the effects of mental and physical health challenges in rural/urban caregivers may differ by socio-demographic characteristics [34,35]. Examining the impact of these intersecting identities on health will help develop a nuanced understanding of the heterogeneity of the caregiver population and unique challenges that different caregivers face.

We consider the study’s limitations below. As with all BRFSS survey items, responses to the mental and physical health questions are self-reported. However, the self-reported nature of the BRFSS survey is countered by findings from CDC- and independent investigator-conducted BRFSS validation studies indicating survey responses show good agreement with in-person measurements and electronic health record information on clinical measures [38]. Additionally, the BRFSS only surveys non-institutionalized individuals aged 18 years or older, and, as a result, our study does not include people less than 18 years providing care to PWD [12]. It is likely that people under 18 years in these caregiving situations would have different challenges affecting their mental and physical health than adult caregivers with such questions warranting a separate study.

## Conclusions

Both rural and urban caregivers of PWD are susceptible to mental health challenges. Rural caregivers are particularly vulnerable to prolonged physical health issues. With the increasing aging trend, caregivers of PWD are expected to provide care for a longer period of time and at a much older age. Therefore, maintaining long-term mental and physical well-being is of great importance to caregivers of PWD. Policy initiatives should aim to enhance access to mental and physical health services tailored to the specific needs of caregivers in different settings. This may involve investing in telehealth services to bridge geographic barriers and provide timely support for rural caregivers. Additionally, prioritizing flexible workplace policies to help urban caregivers balance professional and caregiving responsibilities effectively.

## Supporting information

S1 Table Behavioral Risk Factor Surveillance System survey questions used in study. (DOCX)

S2 Table Backward selection process conserving rural/urban and age, sex, race/ethnicity interactions and interplay between mental and physical health for mental health outcome. (DOCX)

S3 Table Backward selection process conserving rural/urban and age, sex, race/ethnicity interactions and interplay between mental and physical health for physical health outcome. (DOCX)

S4 Table Sensitivity Analysis with non-imputed covariates dataset of Associations between rural/urban residence, sociodemographic factors, and caregiving factors on mental health in previous month comparing outcome of 1–13 poor mental health days vs 0 days. (DOCX)

S5 Table Sensitivity Analysis with non-imputed covariates dataset of Associations between rural/urban residence, sociodemographic factors, and caregiving factors on mental health in previous month comparing outcome of 14 + poor mental health days vs 0 days. (DOCX)

S6 Table Sensitivity Analysis with non-imputed covariates dataset of Associations between rural/urban residence, sociodemographic factors, and caregiving factors on physical health in previous month comparing outcome of 1–13 poor physical health days vs 0 days. (DOCX)

S7 Table Sensitivity Analysis with non-imputed covariates dataset of Associations between rural/urban residence, sociodemographic factors, and caregiving factors on physical health in previous month comparing outcome of 1–13 poor physical health days vs 0 days. (DOCX)
