# Supplementary material for: Mental and physical health of US rural/urban caregivers of persons with dementia
Source: PLoS One. 2025 Aug 1;20(8):e0329260. doi: 10.1371/journal.pone.0329260 (PMC12316319; doi:10.1371/journal.pone.0329260)
Supplement: S1 Table — (DOCX) [file pone.0329260.s001.docx]

**S1 Table.** Behavioral Risk Factor Surveillance System survey questions used in study^[[1]](#endnote-1)^

| Item | BRFSS Question | Responses |
| --- | --- | --- |
| Caregiver status | 2020, 2021, 2022 surveys  "During the past 30 days, did you provide regular care or assistance to a friend or family member who has a health problem or disability?" | 2020, 2021, 2022 surveys  "Yes", "No" |
| Health problem of person providing care for | 2020, 2021, 2022 surveys  "What is the main health problem, long-term illness, or disability that the person you care for has?" | 2020, 2021, 2022 surveys  "Arthritis/ rheumatism", "Asthma", "Cancer", "Chronic respiratory conditions such as emphysema or COPD", "Alzheimer´s disease, dementia or other cognitive impairment disorder", "Developmental disabilities such as autism, Down´s Syndrome, and spina bifida", "Diabetes", "Heart disease, hypertension, stroke", "Human Immunodeficiency Virus Infection (H.I.V.)", "Mental illnesses, such as anxiety, depression, or schizophrenia", "Other organ failure or diseases such as kidney or liver problems", "Substance abuse or addiction disorders", "Injuries, including broken bones", "Old age/infirmity/frailty", "Other"  Note: Reponses of either "Alzheimer´s disease, dementia or other cognitive impairment disorder" to this question or “Yes” to the Caregiver of someone with Alzheimer's disease item question were used to identify caregivers of PWD. |
| Caregiver of someone with Alzheimer's disease | 2020, 2021, 2022 surveys  "Does the person you care for also have Alzheimer´s disease, dementia or other cognitive impairment disorder?" | 2020, 2021, 2022 surveys  "Yes", "No" |
| Rural/Urban status | 2020, 2021, 2022 surveys  "Urban/Rural Status" | 2020, 2021, 2022 surveys  "Urban counties", "Rural counties" |
| Mental health status | 2020, 2021, 2022 surveys  "3 level not good mental health status: 0 days, 1-13 days, 14-30 days" | 2020, 2021, 2022 surveys  "Zero days when mental health not good", "1-13 days when mental health not good", "14+ days when mental health not good" |
| Physical health status | 2020, 2021, 2022 surveys  "3 level not good physical health status: 0 days, 1-13 days, 14-30 days" | 2020, 2021, 2022 surveys  "Zero days when physical health not good", "1-13 days when physical health not good", "14+ days when physical health not good" |
| Age | 2020, 2021, 2022 surveys  "Imputed age category" | 2020, 2021, 2022 surveys  "18-44", "45-64", "65 and older" |
| Sex | 2020, 2021, 2022 surveys  "Sex of Respondent" | 2020, 2021, 2022 surveys  "Male", "Female" |
| Race/Ethnicity | 2020, 2021, 2022 surveys  "Five-level race/ethnicity category" | 2020, 2021, 2022 surveys  "White only, Non-Hispanic", "Black only, Non-Hispanic", "Other race only, Non-Hispanic", "Multiracial, Non-Hispanic", "Hispanic" |
| Household Size | 2020, 2021, 2022 surveys  ***Landline respondents***  **Variable:** NUMADULT  **Question:** I need to randomly select one adult who lives in your household to be interviewed. Excluding adults living away from home, such as students away at college, how many members of your household, including yourself, are 18 years of age or older?  ***Cell phone respondents***  **Variable:** HHADULT  **Question:** How many members of your household, including yourself, are 18 years of age or older?  **Variable:** CHILDREN  **Question:** How many children less than 18 years of age live in your household? | 2020, 2021, 2022 surveys  NUMADULT: 1, 2, 3, 4, 5, 6-99  HHADULT: 0, 1-76  CHILDREN: 1-87 Number of children, None |
| Employment | 2020, 2021, 2022 surveys  Are you currently…? | 2020, 2021, 2022 surveys  Employed for wages, Self-employed, Out of work for 1 year or more, Out of work for less than 1 year, A homemaker, A student, Retired, Unable to work |
| Income | 2020, 2021, 2022 surveys  "Income categories" | 2020, 2021, 2022 surveys  "<$15,000", "$15,000-<$25,000", "$25,000-<$35,000" "$35,000-<$50,000", "$50,000 or more" |
| Education | 2020, 2021, 2022 surveys  "What is the highest grade or year of school you completed?" | 2020, 2021, 2022 surveys  "Did not complete high school", "High school graduate", "Some college or technical school", "College graduate" |
| Health insurance | 2020 survey  “Do you have any kind of health care coverage, including health insurance, prepaid plans such as HMOs, or government plans such as Medicare, or Indian Health Service?”  2021, 2022 surveys  "Adults who had some form of health insurance" | 2020 survey  “Yes”, “No”  2021, 2022 surveys  "Have some form of insurance", "Do not have some form of health insurance" |
| Personal doctor | 2020 survey  “Do you have one person you think of as your personal doctor or health care provider?”  2021, 2022 surveys  "Do you have one person or a group of doctors that you think of as your personal health care provider?" | 2020, 2021, 2022 surveys  "Yes, only one", "More than one", "No" |
| Caregiving relationship of person you are providing care for | 2020, 2021, 2022 surveys  "What is his or her relationship to you?" | 2020, 2021, 2022 surveys  "Child", "Non-relative/Family Friend", "Other relative", "Parent/Parent in law", "Spouse/Live-In partner" |
| Caregiving hours | 2020, 2021, 2022 surveys  "In an average week, how many hours do you provide care or assistance?" | 2020, 2021, 2022 surveys  "Up to 8 hours/week", "9 to 19 hours/week", "20 to 39 hours/week", "40 hours or more/week" |

1. All quotations from this table come from the Centers for Disease Control & Prevention. LLCP 2022 Codebook Report 2023 [October 12, 2024]. Available from: file:///Users/ptran4/Downloads/USCODE22_LLCP_102523.HTML. [↑](#endnote-ref-1)
