# Supplementary material for: Mental and physical health of US rural/urban caregivers of persons with dementia
Source: PLoS One. 2025 Aug 1;20(8):e0329260. doi: 10.1371/journal.pone.0329260 (PMC12316319; doi:10.1371/journal.pone.0329260)
Supplement: S2 Table — (DOCX) [file pone.0329260.s002.docx]

**S2 Table.** Backward selection process conserving rural/urban and age, sex, race/ethnicity interactions and interplay between mental and physical health for mental health outcome

| Step 0. The following effects were entered:  Intercept Physical_Health Rural_Urban Sex Race Income Insurance Personal_Doctor Education Age Household_Size Employmnet Caregiving_Relationship Caregiving_Hours Age*Rural_Urban Sex*Rural_Urban Race*Rural_Urban | \| **Model Fit Statistics** \| \| \| \| --- \| --- \| --- \| \| **Criterion** \| **Intercept Only** \| **Intercept and Covariates** \| \| **AIC** \| 35430.688 \| 31733.175 \| \| **SC** \| 35446.217 \| 32369.865 \| \| **-2 Log L** \| 35426.688 \| 31569.175 \| |
| --- | --- | --- | --- | --- | --- | --- | --- | --- | --- | --- | --- | --- | --- | --- | --- | --- |
| Step 1. Effect Insurance is removed | \| **Model Fit Statistics** \| \| \| \| --- \| --- \| --- \| \| **Criterion** \| **Intercept Only** \| **Intercept and Covariates** \| \| **AIC** \| 35430.688 \| 31729.232 \| \| **SC** \| 35446.217 \| 32350.393 \| \| **-2 Log L** \| 35426.688 \| 31569.232 \| |
| Step 2. Effect Sex*Rural_Urban is removed | \| **Model Fit Statistics** \| \| \| \| --- \| --- \| --- \| \| **Criterion** \| **Intercept Only** \| **Intercept and Covariates** \| \| **AIC** \| 35430.688 \| 31725.637 \| \| **SC** \| 35446.217 \| 32331.269 \| \| **-2 Log L** \| 35426.688 \| 31569.637 \| |
| Step 3. Effect Race*Rural_Urban is removed | \| **Model Fit Statistics** \| \| \| \| --- \| --- \| --- \| \| **Criterion** \| **Intercept Only** \| **Intercept and Covariates** \| \| **AIC** \| 35430.688 \| 31721.716 \| \| **SC** \| 35446.217 \| 32265.232 \| \| **-2 Log L** \| 35426.688 \| 31581.716 \| |
| **Step 4. Effect Age***Rural_Urban **is removed** | \| **Model Fit Statistics** \| \| \| \| --- \| --- \| --- \| \| **Criterion** \| **Intercept Only** \| **Intercept and Covariates** \| \| **AIC** \| 35430.688 \| 31721.895 \| \| **SC** \| 35446.217 \| 32234.353 \| \| **-2 Log L** \| 35426.688 \| 31589.895 \| |
| Step 5. Effect Race is removed | \| **Model Fit Statistics** \| \| \| \| --- \| --- \| --- \| \| **Criterion** \| **Intercept Only** \| **Intercept and Covariates** \| \| **AIC** \| 35430.688 \| 31720.303 \| \| **SC** \| 35446.217 \| 32170.645 \| \| **-2 Log L** \| 35426.688 \| 31604.303 \| |
