# Supplementary material for: Mental and physical health of US rural/urban caregivers of persons with dementia
Source: PLoS One. 2025 Aug 1;20(8):e0329260. doi: 10.1371/journal.pone.0329260 (PMC12316319; doi:10.1371/journal.pone.0329260)
Supplement: S3 Table — (DOCX) [file pone.0329260.s003.docx]

**S3 Table.** Backward selection process conserving rural/urban and age, sex, race/ethnicity interactions and interplay between mental and physical health for physical health outcome

| Step 0. The following effects were entered:  Intercept Mental_Health Rural_Urban Sex Race Income Insurance Personal_Doctor Education Age Household_Size Employmnet Caregiving_Relationship Caregiving_Hours Age*Rural_Urban Sex*Rural_Urban Race*Rural_Urban | \| **Model Fit Statistics** \| \| \| \| --- \| --- \| --- \| \| **Criterion** \| **Intercept Only** \| **Intercept and Covariates** \| \| **AIC** \| 32747.373 \| 29510.030 \| \| **SC** \| 32762.902 \| 30146.720 \| \| **-2 Log L** \| 32743.373 \| 29346.030 \| |
| --- | --- | --- | --- | --- | --- | --- | --- | --- | --- | --- | --- | --- | --- | --- | --- | --- |
| Step 1. Effect Sex*Rural_Urban is removed | \| **Model Fit Statistics** \| \| \| \| --- \| --- \| --- \| \| **Criterion** \| **Intercept Only** \| **Intercept and Covariates** \| \| **AIC** \| 32747.373 \| 29506.227 \| \| **SC** \| 32762.902 \| 30127.388 \| \| **-2 Log L** \| 32743.373 \| 29346.227 \| |
| Step 2. Effect Race*Rural_Urban is removed | \| **Model Fit Statistics** \| \| \| \| --- \| --- \| --- \| \| **Criterion** \| **Intercept Only** \| **Intercept and Covariates** \| \| **AIC** \| 32747.373 \| 29494.435 \| \| **SC** \| 32762.902 \| 30053.480 \| \| **-2 Log L** \| 32743.373 \| 29350.435 \| |
| Step 3. Effect Household_Size is removed | \| **Model Fit Statistics** \| \| \| \| --- \| --- \| --- \| \| **Criterion** \| **Intercept Only** \| **Intercept and Covariates** \| \| **AIC** \| 32747.373 \| 29488.444 \| \| **SC** \| 32762.902 \| 30016.431 \| \| **-2 Log L** \| 32743.373 \| 29352.444 \| |
| Step 4. Effect Insurance is removed | \| **Model Fit Statistics** \| \| \| \| --- \| --- \| --- \| \| **Criterion** \| **Intercept Only** \| **Intercept and Covariates** \| \| **AIC** \| 32747.373 \| 29485.074 \| \| **SC** \| 32762.902 \| 29997.532 \| \| **-2 Log L** \| 32743.373 \| 29353.074 \| |
| Step 5. Effect Race is removed | \| **Model Fit Statistics** \| \| \| \| --- \| --- \| --- \| \| **Criterion** \| **Intercept Only** \| **Intercept and Covariates** \| \| **AIC** \| 32747.373 \| 29478.500 \| \| **SC** \| 32762.902 \| 29928.841 \| \| **-2 Log L** \| 32743.373 \| 29362.500 \| |
| Step 6. Effect Age*Rural_Urban is removed | \| **Model Fit Statistics** \| \| \| \| --- \| --- \| --- \| \| **Criterion** \| **Intercept Only** \| **Intercept and Covariates** \| \| **AIC** \| 32747.373 \| 29476.889 \| \| **SC** \| 32762.902 \| 29896.172 \| \| **-2 Log L** \| 32743.373 \| 29368.889 \| |
