# Supplementary material for: Mental and physical health of US rural/urban caregivers of persons with dementia
Source: PLoS One. 2025 Aug 1;20(8):e0329260. doi: 10.1371/journal.pone.0329260 (PMC12316319; doi:10.1371/journal.pone.0329260)
Supplement: S4 Table — (DOCX) [file pone.0329260.s004.docx]

**S4 Table.** Sensitivity Analysis with non-imputed covariates dataset of Associations between rural/urban residence, sociodemographic factors, and caregiving factors on mental health in previous month comparing outcome of 1-13 poor mental health days vs 0 days

| Covariate | Unadjusted Model^[[1]](#footnote-1)^ | | Adjusted Model  (sociodemographic factors not imputed)^[[2]](#footnote-2)^ | | Adjusted Model (sociodemographic and caregiving factors not imputed)^[[3]](#footnote-3)^ | | Adjusted Model (sociodemographic and caregiving factors not imputed) with covariates from backwards selection^[[4]](#footnote-4)^ | |
| --- | --- | --- | --- | --- | --- | --- | --- | --- |
|  | Odds Ratio | P-value | Odds Ratio | P-value | Odds Ratio | P-value^[[5]](#footnote-5)^ | Odds Ratio | P-value |
|  | (1-13 days when mental health not good vs 0 days when mental health not good) |  | (1-13 days when mental health not good vs 0 days when mental health not good) |  | (1-13 days when mental health not good vs 0 days when mental health not good) |  | (1-13 days when mental health not good vs 0 days when mental health not good) |  |
| **Rural/Urban status**  **(ref: Urban)** |  | 0.0361 |  | 0.025 |  | 0.0179 |  | 0.0247 |
| Rural^[[6]](#footnote-6)^ | 0.59 (0.34, 1.05) |  | 0.45 (0.25, 0.82)* |  | 0.43 (0.23, 0.80)* |  | 0.45 (0.25, 0.81)* |  |
| **Age**  **(ref: 18-44)** |  | -- |  | 0.0198 |  | 0.0182 |  | 0.0001 |
| 45-64 | -- |  | 0.54 (0.27, 1.06) |  | 0.55 (0.28, 1.06) |  | 0.59 (0.3, 1.13) |  |
| 65 and older | -- |  | 0.39 (0.17, 0.87)* |  | 0.51 (0.22, 1.17) |  | 0.58 (0.26, 1.3) |  |
| **Sex**  **(ref: Male)** |  | -- |  | 0.1926 |  | 0.1527 |  | 0.124 |
| Female | -- |  | 1.2 (0.74, 1.93) |  | 1.25 (0.80, 1.95) |  | 1.16 (0.72, 1.85) |  |
| **Race/Ethnicity**  **(ref: White only, Non-Hispanic)** |  | -- |  | 0.0034 |  | 0.0044 |  | 0.0024 |
| Black only, Non-Hispanic | -- |  | 0.96 (0.45, 2.05) |  | 1.01 (0.49, 2.10) |  | 0.90 (0.43, 1.89) |  |
| Other race only, Non-Hispanic | -- |  | 0.66 (0.35, 1.25) |  | 0.64 (0.35, 1.18) |  | 0.64 (0.33, 1.24) |  |
| Multiracial, Non-Hispanic | -- |  | 6.48 (1.25, 33.63)* |  | 5.88 (1.45, 23.9)* |  | 8.42 (1.65, 42.99)* |  |
| Hispanic | -- |  | 0.54 (0.2, 1.41) |  | 0.67 (0.30, 1.48) |  | 0.75 (0.35, 1.62) |  |
| **Household Size**  **(ref: 1 person)** |  | -- |  | 0.177 |  | 0.5415 |  | 0.2045 |
| 2-4 people | -- |  | 1.64 (0.76, 3.55) |  | 1.75 (0.79, 3.89) |  | 1.66 (0.80, 3.48) |  |
| >4 people | -- |  | 1.79 (0.69, 4.62) |  | 1.97 (0.74, 5.25) |  | 2.02 (0.85, 4.79) |  |
| **Employment**  **(ref: Employed for wages)** |  | -- |  | 0.0634 |  | 0.2282 |  | 0.3366 |
| Self-employed | -- |  | 0.62 (0.22, 1.73) |  | 0.62 (0.24, 1.60) |  | 0.70 (0.29, 1.67) |  |
| Out of work for 1 year or more | -- |  | 1.36 (0.63, 2.96) |  | 1.29 (0.58, 2.88) |  | 1.58 (0.67, 3.74) |  |
| Out of work for < 1 year | -- |  | 0.41 (0.15, 1.13) |  | 0.44 (0.17, 1.15) |  | 0.46 (0.18, 1.20) |  |
| Out of the work force (includes homemaker, a student, retired, unable to work) | -- |  | 1.00 (0.57, 1.75) |  | 0.95 (0.54, 1.64) |  | 0.96 (0.54, 1.72) |  |
| **Education**  **(ref: College graduate)** |  | -- |  | 0.189 |  | 0.0137 |  | 0.0128 |
| Did not complete high school | -- |  | 0.51 (0.28, 0.92)* |  | 0.48 (0.25, 0.92)* |  | 0.43 (0.23, 0.82)* |  |
| High school graduate | -- |  | 0.78 (0.41, 1.49) |  | 0.81 (0.42, 1.54) |  | 0.75 (0.37, 1.49) |  |
| Some college or technical school | -- |  | 0.86 (0.52, 1.44) |  | 0.90 (0.57, 1.43) |  | 0.84 (0.54, 1.31) |  |
| **Income**  **(ref: <$15,000)** |  | -- |  | <.0001 |  | <.0001 |  | 0.012 |
| $15,000-<$25,000 | -- |  | 0.80 (0.32, 1.99) |  | 0.78 (0.32, 1.88) |  | 0.79 (0.33, 1.89) |  |
| $25,000-<$35,000 | -- |  | 0.94 (0.39, 2.27) |  | 0.97 (0.41, 2.31) |  | 1.06 (0.45, 2.51) |  |
| $35,000-<$50,000 | -- |  | 0.35 (0.14, 0.88)* |  | 0.37 (0.15, 0.93)* |  | 0.40 (0.16, 1.05) |  |
| $50,000 or more | -- |  | 0.51 (0.22, 1.14) |  | 0.51 (0.23, 1.15) |  | 0.61 (0.27, 1.39) |  |
| **Health Insurance**  **(ref: No)** |  | -- |  | 0.7802 |  | 0.5264 |  | 0.239 |
| Yes | -- |  | 1.09 (0.54, 2.20) |  | 1.10 (0.56, 2.18) |  | 1.02 (0.53, 1.96) |  |
| **Personal Doctor**  **(ref: No)** |  | -- |  | 0.146 |  | 0.0763 |  | 0.1081 |
| Yes, only one | -- |  | 0.96 (0.45, 2.04) |  | 0.95 (0.45, 2.02) |  | 1.05 (0.47, 2.35) |  |
| More than one | -- |  | 0.58 (0.26, 1.31) |  | 0.58 (0.25, 1.33) |  | 0.55 (0.23, 1.34) |  |
| **Caregiving Relationship**  **(ref: Non-relative/Family Friend)** |  | -- |  | -- |  | 0.3337 |  | 0.5084 |
| Child | -- |  | -- |  | 1.50 (0.58, 3.85) |  | 1.37 (0.55, 3.37) |  |
| Other relative | -- |  | -- |  | 0.90 (0.37, 2.21) |  | 0.86 (0.34, 2.18) |  |
| Parent/Parent in law | -- |  | -- |  | 1.31 (0.63, 2.71) |  | 1.16 (0.56, 2.40) |  |
| Spouse/Live-In partner | -- |  | -- |  | 0.61 (0.25, 1.47) |  | 0.60 (0.25, 1.43) |  |
| **Caregiving Hours**  **(ref: Up to 8 hours/week)** |  | -- |  | -- |  | 0.0128 |  | 0.0084 |
| 9 to 19 hours/week | -- |  | -- |  | 1.41 (0.76, 2.64) |  | 1.30 (0.68, 2.49) |  |
| 20 to 39 hours/week | -- |  | -- |  | 0.53 (0.24, 1.17) |  | 0.51 (0.22, 1.15) |  |
| 40 hours or more/week | -- |  | -- |  | 1.29 (0.70, 2.36) |  | 1.19 (0.64, 2.2) |  |
| **Physical Health**  **(ref: 0 days)** |  | -- |  | -- |  | -- |  | <.0001 |
| 1-13 days | -- |  | -- |  | -- |  | 3.20 (1.89, 5.43)* |  |
| 14+ days | -- |  | -- |  | -- |  | 1.84 (0.88, 3.86) |  |

1. Model includes rural/urban status only. [↑](#footnote-ref-1)
2. Model covariates include rural/urban status and sociodemographic factors (age, sex, race, education, household size, employment, income, health insurance, personal doctor). [↑](#footnote-ref-2)
3. Model covariates include rural/urban status, sociodemographic factors (age, sex, race/ethnicity, household size, employment, education, income, health insurance, personal doctor), and caregiving factors (caregiving relationship, caregiving hours). [↑](#footnote-ref-3)
4. Model covariates include rural/urban status, sociodemographic factors (age, sex, race/ethnicity, household size, employment, education, income, health insurance, personal doctor), caregiving factors (caregiving relationship, caregiving hours), and physical health. [↑](#footnote-ref-4)
5. For a multinomial logistic model, the overall Chi-squared test p value for a variable (i.e., race, sex education) will be the same for a variable comparing 1-13 poor mental health days vs 0 days (S4 Table) and comparing 14+ poor mental health days vs 0 days (S5 Table). [↑](#footnote-ref-5)
6. An asterisk (*) indicates that a category is statistically significant (p-value<0.05) from the reference category in terms of the outcome. [↑](#footnote-ref-6)
