# Supplementary material for: Mental and physical health of US rural/urban caregivers of persons with dementia
Source: PLoS One. 2025 Aug 1;20(8):e0329260. doi: 10.1371/journal.pone.0329260 (PMC12316319; doi:10.1371/journal.pone.0329260)
Supplement: S5 Table — (DOCX) [file pone.0329260.s005.docx]

**S5 Table.** Sensitivity Analysis with non-imputed covariates dataset of Associations between rural/urban residence, sociodemographic factors, and caregiving factors on mental health in previous month comparing outcome of 14+ poor mental health days vs 0 days

| Covariate | Unadjusted Model^[[1]](#footnote-1)^ | | Adjusted Model  (sociodemographic factors not imputed)^[[2]](#footnote-2)^ | | Adjusted Model (sociodemographic and caregiving factors not imputed)^[[3]](#footnote-3)^ | | Adjusted Model (sociodemographic and caregiving factors not imputed) with covariates from backwards selection^[[4]](#footnote-4)^ | |
| --- | --- | --- | --- | --- | --- | --- | --- | --- |
|  | Odds Ratio | P-value | Odds Ratio | P-value | Odds Ratio | P-value^[[5]](#footnote-5)^ | Odds Ratio | P-value |
|  | (14+ days when mental health not good vs 0 days when mental health not good) |  | (14+ days when mental health not good vs 0 days when mental health not good) |  | (14+ days when mental health not good vs 0 days when mental health not good) |  | (14+ days when mental health not good vs 0 days when mental health not good) |  |
| **Rural/Urban status**  **(ref: Urban)** |  | 0.0361 |  | 0.025 |  | 0.0179 |  | 0.0247 |
| Rural^[[6]](#footnote-6)^ | 1.13 (0.55, 2.30) |  | 0.78 (0.37, 1.63) |  | 0.82 (0.38, 1.78) |  | 0.63 (0.24, 1.63) |  |
| **Age**  **(ref: 18-44)** |  | -- |  | 0.0198 |  | 0.0182 |  | 0.0001 |
| 45-64 | -- |  | 0.54 (0.28, 1.07) |  | 0.48 (0.26, 0.88)* |  | 0.41 (0.22, 0.78)* |  |
| 65 and older | -- |  | 0.28 (0.13, 0.60)* |  | 0.26 (0.11, 0.57)* |  | 0.38 (0.17, 0.84)* |  |
| **Sex**  **(ref: Male)** |  | -- |  | 0.1926 |  | 0.1527 |  | 0.124 |
| Female | -- |  | 1.62 (0.96, 2.71) |  | 1.66 (0.99, 2.77) |  | 1.84 (1.02, 3.30)* |  |
| **Race/Ethnicity**  **(ref: White only, Non-Hispanic)** |  | -- |  | 0.0034 |  | 0.0044 |  | 0.0024 |
| Black only, Non-Hispanic | -- |  | 0.65 (0.28, 1.51) |  | 0.61 (0.27, 1.41) |  | 0.55 (0.24, 1.25) |  |
| Other race only, Non-Hispanic | -- |  | 2.34 (0.90, 6.09) |  | 0.97 (0.43, 2.17) |  | 0.96 (0.39, 2.40) |  |
| Multiracial, Non-Hispanic | -- |  | 4.94 (1.77, 13.81)* |  | 4.60 (1.76, 12.03)* |  | 5.41 (2.01, 14.53)* |  |
| Hispanic | -- |  | 0.48 (0.22, 1.04) |  | 0.41 (0.17, 0.98)* |  | 0.39 (0.17, 0.89)* |  |
| **Household Size**  **(ref: 1 person)** |  | -- |  | 0.177 |  | 0.5415 |  | 0.2045 |
| 2-4 people | -- |  | 1.69 (0.90, 3.19) |  | 1.68 (0.82, 3.44) |  | 2.00 (0.97, 4.11) |  |
| >4 people | -- |  | 3.13 (1.21, 8.11)* |  | 1.92 (0.71, 5.19) |  | 2.85 (1.10, 7.4)* |  |
| **Employment**  **(ref: Employed for wages)** |  | -- |  | 0.0634 |  | 0.2282 |  | 0.3366 |
| Self-employed | -- |  | 0.69 (0.30, 1.55) |  | 0.78 (0.36, 1.66) |  | 0.82 (0.41, 1.62) |  |
| Out of work for 1 year or more | -- |  | 1.95 (0.82, 4.65) |  | 2.19 (0.86, 5.57) |  | 2.42 (0.88, 6.62) |  |
| Out of work for < 1 year | -- |  | 0.27 (0.07, 1.09) |  | 0.27 (0.06, 1.21) |  | 0.16 (0.03, 1.02) |  |
| Out of the work force (includes homemaker, a student, retired, unable to work) | -- |  | 1.71 (0.90, 3.25) |  | 1.43 (0.75, 2.74) |  | 1.04 (0.56, 1.93) |  |
| **Education**  **(ref: College graduate)** |  | -- |  | 0.189 |  | 0.0137 |  | 0.0128 |
| Did not complete high school | -- |  | 1.09 (0.43, 2.78) |  | 1.18 (0.48, 2.94) |  | 0.90 (0.37, 2.21) |  |
| High school graduate | -- |  | 0.65 (0.34, 1.23) |  | 0.48 (0.26, 0.89)* |  | 0.43 (0.23, 0.83)* |  |
| Some college or technical school | -- |  | 1.10 (0.54, 2.23) |  | 0.90 (0.48, 1.67) |  | 0.87 (0.46, 1.64) |  |
| **Income**  **(ref: <$15,000)** |  | -- |  | <.0001 |  | <.0001 |  | 0.012 |
| $15,000-<$25,000 | -- |  | 0.97 (0.44, 2.14) |  | 0.70 (0.31, 1.57) |  | 0.78 (0.33, 1.85) |  |
| $25,000-<$35,000 | -- |  | 0.72 (0.31, 1.65) |  | 0.83 (0.35, 1.94) |  | 0.92 (0.35, 2.43) |  |
| $35,000-<$50,000 | -- |  | 0.25 (0.11, 0.57)* |  | 0.24 (0.10, 0.55)* |  | 0.34 (0.14, 0.84)* |  |
| $50,000 or more | -- |  | 0.16 (0.08, 0.33)* |  | 0.16 (0.07, 0.34)* |  | 0.24 (0.10, 0.55)* |  |
| **Health Insurance**  **(ref: No)** |  | -- |  | 0.7802 |  | 0.5264 |  | 0.239 |
| Yes | -- |  | 0.84 (0.41, 1.72) |  | 0.74 (0.35, 1.57) |  | 0.60 (0.29, 1.21) |  |
| **Personal Doctor**  **(ref: No)** |  | -- |  | 0.146 |  | 0.0763 |  | 0.1081 |
| Yes, only one | -- |  | 0.59 (0.24, 1.43) |  | 0.67 (0.31, 1.47) |  | 0.71 (0.30, 1.66) |  |
| More than one | -- |  | 0.73 (0.28, 1.86) |  | 0.92 (0.39, 2.17) |  | 0.68 (0.27, 1.72) |  |
| **Caregiving Relationship**  **(ref: Non-relative/Family Friend)** |  | -- |  | -- |  | 0.3337 |  | 0.5084 |
| Child | -- |  | -- |  | 1.50 (0.61, 3.68) |  | 1.22 (0.52, 2.85) |  |
| Other relative | -- |  | -- |  | 1.11 (0.50, 2.48) |  | 1.27 (0.59, 2.71) |  |
| Parent/Parent in law | -- |  | -- |  | 1.00 (0.48, 2.09) |  | 0.98 (0.48, 2.00) |  |
| Spouse/Live-In partner | -- |  | -- |  | 1.01 (0.40, 2.58) |  | 0.87 (0.36, 2.15) |  |
| **Caregiving Hours**  **(ref: Up to 8 hours/week)** |  | -- |  | -- |  | 0.0128 |  | 0.0084 |
| 9 to 19 hours/week | -- |  | -- |  | 1.08 (0.53, 2.20) |  | 1.12 (0.51, 2.45) |  |
| 20 to 39 hours/week | -- |  | -- |  | 2.38 (1.06, 5.3)* |  | 2.74 (1.23, 6.11)* |  |
| 40 hours or more/week | -- |  | -- |  | 1.28 (0.70, 2.35) |  | 1.16 (0.62, 2.18) |  |
| **Physical Health**  **(ref: 0 days)** |  | -- |  | -- |  | -- |  | <.0001 |
| 1-13 days | -- |  | -- |  | -- |  | 2.77 (1.32, 5.81)* |  |
| 14+ days | -- |  | -- |  | -- |  | 10.58 (5.40, 20.73)* |  |

1. Model includes rural/urban status only. [↑](#footnote-ref-1)
2. Model covariates include rural/urban status and sociodemographic factors (age, sex, race/ethnicity, household size, employment, education, income, health insurance, personal doctor). [↑](#footnote-ref-2)
3. Model covariates include rural/urban status and sociodemographic factors (age, sex, race/ethnicity, household size, employment, education, income, health insurance, personal doctor).and caregiving factors (caregiving relationship, caregiving hours). [↑](#footnote-ref-3)
4. Model covariates include rural/urban status, sociodemographic factors (age, sex, race/ethnicity, household size, employment, education, income, health insurance, personal doctor), caregiving factors (caregiving relationship, caregiving hours), and physical health. [↑](#footnote-ref-4)
5. For a multinomial logistic model, the overall Chi-squared test p value for a variable (i.e., race, sex education) will be the same for a variable comparing 1-13 poor mental health days vs 0 days (S4 Table) and comparing 14+ poor mental health days vs 0 days (S5 Table). [↑](#footnote-ref-5)
6. An asterisk (*) indicates that a category is statistically significant (p-value<0.05) from the reference category in terms of the outcome. [↑](#footnote-ref-6)
