# Supplementary material for: Mental and physical health of US rural/urban caregivers of persons with dementia
Source: PLoS One. 2025 Aug 1;20(8):e0329260. doi: 10.1371/journal.pone.0329260 (PMC12316319; doi:10.1371/journal.pone.0329260)
Supplement: S6 Table — (DOCX) [file pone.0329260.s006.docx]

**S6 Table.** Sensitivity Analysis with non-imputed covariates dataset of Associations between rural/urban residence, sociodemographic factors, and caregiving factors on physical health in previous month comparing outcome of 1-13 poor physical health days vs 0 days

| Covariate | Unadjusted Model^[[1]](#footnote-1)^ | | Adjusted Model  (sociodemographic factors)^[[2]](#footnote-2)^ | | Adjusted Model (sociodemographic and caregiving factors)^[[3]](#footnote-3)^ | | Adjusted Model (sociodemographic and caregiving factors) with covariates from backwards selection^[[4]](#footnote-4)^ | |
| --- | --- | --- | --- | --- | --- | --- | --- | --- |
|  | Odds Ratio | P-value | Odds Ratio | P-value | Odds Ratio | P-value^[[5]](#footnote-5)^ | Odds Ratio | P-value |
|  | (1-13 days when physical health not good vs 0 days when physical health not good) |  | (1-13 days when physical health not good vs 0 days when physical health not good) |  | (1-13 days when physical health not good vs 0 days when physical health not good) |  | (1-13 days when physical health not good vs 0 days when physical health not good) |  |
| **Rural/Urban status**  **(ref: Urban)** |  | 0.0734 |  | 0.0222 |  | 0.0104 |  | 0.0114 |
| Rural^[[6]](#footnote-6)^ | 0.86 (0.53, 1.41) |  | 0.75 (0.39, 1.43) |  | 0.76 (0.37, 1.54) |  | 0.88 (0.43, 1.81) |  |
| **Age**  **(ref: 18-44)** |  | -- |  | <.0001 |  | <.0001 |  | 0.0001 |
| 45-64 | -- |  | 0.81 (0.43, 1.54) |  | 0.69 (0.37, 1.28) |  | 0.83 (0.45, 1.53) |  |
| 65 and older | -- |  | 0.45 (0.21, 0.95) |  | 0.56 (0.26, 1.19) |  | 0.67 (0.32, 1.42) |  |
| **Sex**  **(ref: Male)** |  | -- |  | 0.3221 |  | 0.2551 |  | 0.1617 |
| Female | -- |  | 1.18 (0.71, 1.98) |  | 1.36 (0.81, 2.28) |  | 1.29 (0.75, 2.22) |  |
| **Race/Ethnicity**  **(ref: White only, Non-Hispanic)** |  | -- |  | 0.0078 |  | 0.0416 |  | 0.0637 |
| Black only, Non-Hispanic | -- |  | 1.42 (0.66, 3.03) |  | 1.46 (0.73, 2.94) |  | 1.50 (0.73, 3.08) |  |
| Other race only, Non-Hispanic | -- |  | 2.26 (0.86, 6.00) |  | 0.86 (0.39, 1.89) |  | 0.92 (0.40, 2.15) |  |
| Multiracial, Non-Hispanic | -- |  | 0.30 (0.08, 1.11) |  | 0.21 (0.04, 1.01) |  | 0.14 (0.02, 0.84)* |  |
| Hispanic | -- |  | 0.53 (0.24, 1.17) |  | 0.50 (0.25, 1.03) |  | 0.56 (0.30, 1.08) |  |
| **Household Size**  **(ref: 1 person)** |  | -- |  | 0.1395 |  | 0.2323 |  | 0.1599 |
| 2-4 people | -- |  | 1.34 (0.66, 2.74) |  | 1.31 (0.64, 2.69) |  | 1.13 (0.59, 2.18) |  |
| >4 people | -- |  | 1.08 (0.39, 3.02) |  | 0.86 (0.29, 2.54) |  | 0.69 (0.25, 1.93) |  |
| **Employment**  **(ref: Employed for wages)** |  | -- |  | <.0001 |  | <.0001 |  | <.0001 |
| Self-employed | -- |  | 0.55 (0.22, 1.41) |  | 0.49 (0.20, 1.21) |  | 0.53 (0.24, 1.17) |  |
| Out of work for 1 year or more | -- |  | 0.37 (0.17, 0.83)* |  | 0.34 (0.15, 0.80)* |  | 0.30 (0.12, 0.75)* |  |
| Out of work for < 1 year | -- |  | 0.50 (0.21, 1.16) |  | 0.51 (0.21, 1.25) |  | 0.57 (0.25, 1.29) |  |
| Out of the work force (includes homemaker, a student, retired, unable to work) | -- |  | 0.95 (0.54, 1.67) |  | 0.87 (0.50, 1.49) |  | 0.86 (0.49, 1.50) |  |
| **Education**  **(ref: College graduate)** |  | -- |  | 0.1317 |  | 0.0809 |  | 0.101 |
| Did not complete high school | -- |  | 1.18 (0.53, 2.64) |  | 1.32 (0.58, 3.01) |  | 1.52 (0.69, 3.33) |  |
| High school graduate | -- |  | 1.46 (0.79, 2.71) |  | 1.37 (0.76, 2.46) |  | 1.49 (0.80, 2.78) |  |
| Some college or technical school | -- |  | 1.23 (0.68, 2.23) |  | 1.25 (0.75, 2.09) |  | 1.27 (0.75, 2.14) |  |
| **Income**  **(ref: <$15,000)** |  | -- |  | <.0001 |  | <.0001 |  | 0.0015 |
| $15,000-<$25,000 | -- |  | 1.20 (0.49, 2.91) |  | 0.92 (0.38, 2.21) |  | 1.03 (0.42, 2.50) |  |
| $25,000-<$35,000 | -- |  | 0.70 (0.29, 1.70) |  | 0.60 (0.24, 1.50) |  | 0.60 (0.24, 1.48) |  |
| $35,000-<$50,000 | -- |  | 0.59 (0.24, 1.48) |  | 0.52 (0.21, 1.30) |  | 0.65 (0.25, 1.65) |  |
| $50,000 or more | -- |  | 0.47 (0.21, 1.05) |  | 0.42 (0.18, 0.97)* |  | 0.50 (0.22, 1.15) |  |
| **Health Insurance**  **(ref: No)** |  | -- |  | 0.3402 |  | 0.4226 |  | 0.3367 |
| Yes | -- |  | 1.25 (0.61, 2.6) |  | 1.33 (0.63, 2.80) |  | 1.35 (0.65, 2.78) |  |
| **Personal Doctor**  **(ref: No)** |  | -- |  | <.0001 |  | <.0001 |  | <.0001 |
| Yes, only one | -- |  | 1.12 (0.48, 2.63) |  | 0.77 (0.34, 1.74) |  | 0.77 (0.32, 1.83) |  |
| More than one | -- |  | 1.39 (0.56, 3.48) |  | 1.04 (0.43, 2.50) |  | 1.17 (0.45, 3.01) |  |
| **Caregiving Relationship**  **(ref: Non-relative/Family Friend)** |  | -- |  | -- |  | 0.0226 |  | 0.0293 |
| Child | -- |  | -- |  | 1.33 (0.52, 3.41) |  | 1.26 (0.50, 3.16) |  |
| Other relative | -- |  | -- |  | 1.25 (0.56, 2.78) |  | 1.26 (0.54, 2.92) |  |
| Parent/Parent in law | -- |  | -- |  | 1.76 (0.86, 3.61) |  | 1.67 (0.78, 3.56) |  |
| Spouse/Live-In partner | -- |  | -- |  | 0.85 (0.33, 2.19) |  | 0.92 (0.36, 2.33) |  |
| **Caregiving Hours**  **(ref: Up to 8 hours/week)** |  | -- |  | -- |  | 0.2849 |  | 0.2168 |
| 9 to 19 hours/week | -- |  | -- |  | 1.56 (0.83, 2.93) |  | 1.47 (0.76, 2.83) |  |
| 20 to 39 hours/week | -- |  | -- |  | 1.00 (0.39, 2.56) |  | 1.04 (0.39, 2.76) |  |
| 40 hours or more/week | -- |  | -- |  | 1.51 (0.84, 2.72) |  | 1.47 (0.80, 2.73) |  |
| **Mental Health**  **(ref: 0 days)** |  | -- |  | -- |  | -- |  | <.0001 |
| 1-13 days | -- |  | -- |  | -- |  | 3.16 (1.83, 5.46)* |  |
| 14+ days | -- |  | -- |  | -- |  | 2.43 (1.08, 5.45)* |  |

1. Model includes rural/urban status only. [↑](#footnote-ref-1)
2. Model covariates include rural/urban status and sociodemographic factors (age, sex, race/ethnicity, household size, employment, education, income, health insurance, personal doctor). [↑](#footnote-ref-2)
3. Model covariates include rural/urban status and sociodemographic factors (age, sex, race/ethnicity, household size, employment, education, income, health insurance, personal doctor).and caregiving factors (caregiving relationship, caregiving hours). [↑](#footnote-ref-3)
4. Model covariates include rural/urban status, sociodemographic factors (age, sex, race/ethnicity, household size, employment, education, income, health insurance, personal doctor), caregiving factors (caregiving relationship, caregiving hours), and mental health. [↑](#footnote-ref-4)
5. For a multinomial logistic model, the overall Chi-squared test p value for a variable (i.e., race, sex education) will be the same for a variable comparing 1-13 poor physical health days vs 0 days (S6 Table) and comparing 14+ poor physical health days vs 0 days (S7 Table). [↑](#footnote-ref-5)
6. An asterisk (*) indicates that a category is statistically significant (p-value<0.05) from the reference category in terms of the outcome. [↑](#footnote-ref-6)
