# Supplementary material for: Mental and physical health of US rural/urban caregivers of persons with dementia
Source: PLoS One. 2025 Aug 1;20(8):e0329260. doi: 10.1371/journal.pone.0329260 (PMC12316319; doi:10.1371/journal.pone.0329260)
Supplement: S7 Table — (DOCX) [file pone.0329260.s007.docx]

**S7 Table.** Sensitivity Analysis with non-imputed covariates dataset of Associations between rural/urban residence, sociodemographic factors, and caregiving factors on physical health in previous month comparing outcome of 1-13 poor physical health days vs 0 days

| Covariate | Unadjusted Model^[[1]](#footnote-1)^ | | Adjusted Model  (sociodemographic factors)^[[2]](#footnote-2)^ | | Adjusted Model (sociodemographic and caregiving factors)^[[3]](#footnote-3)^ | | Adjusted Model (sociodemographic and caregiving factors) with covariates from backwards selection^[[4]](#footnote-4)^ | |
| --- | --- | --- | --- | --- | --- | --- | --- | --- |
|  | Odds Ratio | P-value | Odds Ratio | P-value | Odds Ratio | P-value^[[5]](#footnote-5)^ | Odds Ratio | P-value |
|  | (14+ days when physical health not good vs 0 days when physical health not good) |  | (14+ days when physical health not good vs 0 days when physical health not good) |  | (14+ days when physical health not good vs 0 days when physical health not good) |  | (14+ days when physical health not good vs 0 days when physical health not good) |  |
| **Rural/Urban status**  **(ref: Urban)** |  | 0.0734 |  | 0.0222 |  | 0.0104 |  | 0.0114 |
| Rural^[[6]](#footnote-6)^ | 2.57 (1.00, 6.63) |  | 2.05 (0.96, 4.36) |  | 2.33 (1.06, 5.13)* |  | 2.65 (1.12, 6.28)* |  |
| **Age**  **(ref: 18-44)** |  | -- |  | <.0001 |  | <.0001 |  | 0.0001 |
| 45-64 | -- |  | 2.17 (1.18, 3.99)* |  | 1.55 (0.92, 2.60) |  | 1.98 (1.13, 3.49)* |  |
| 65 and older | -- |  | 0.54 (0.22, 1.33) |  | 0.26 (0.12, 0.55)* |  | 0.37 (0.16, 0.85)* |  |
| **Sex**  **(ref: Male)** |  | -- |  | 0.3221 |  | 0.2551 |  | 0.1617 |
| Female | -- |  | 0.79 (0.5, 1.25) |  | 0.86 (0.53, 1.37) |  | 0.73 (0.44, 1.23) |  |
| **Race/Ethnicity**  **(ref: White only, Non-Hispanic)** |  | -- |  | 0.0078 |  | 0.0416 |  | 0.0637 |
| Black only, Non-Hispanic | -- |  | 1.16 (0.58, 2.3) |  | 1.15 (0.58, 2.27) |  | 1.45 (0.74, 2.87) |  |
| Other race only, Non-Hispanic | -- |  | 0.74 (0.31, 1.79) |  | 1.10 (0.52, 2.32) |  | 1.10 (0.47, 2.61) |  |
| Multiracial, Non-Hispanic | -- |  | 1.22 (0.38, 3.94) |  | 1.36 (0.44, 4.26) |  | 0.82 (0.26, 2.59) |  |
| Hispanic | -- |  | 2.15 (0.83, 5.56) |  | 1.13 (0.57, 2.24) |  | 1.36 (0.74, 2.50) |  |
| **Household Size**  **(ref: 1 person)** |  | -- |  | 0.1395 |  | 0.2323 |  | 0.1599 |
| 2-4 people | -- |  | 0.96 (0.50, 1.86) |  | 0.74 (0.38, 1.42) |  | 0.63 (0.32, 1.23) |  |
| >4 people | -- |  | 0.43 (0.18, 1.04) |  | 0.43 (0.18, 1.02) |  | 0.36 (0.15, 0.84)* |  |
| **Employment**  **(ref: Employed for wages)** |  | -- |  | <.0001 |  | <.0001 |  | <.0001 |
| Self-employed | -- |  | 1.44 (0.55, 3.8) |  | 0.84 (0.37, 1.94) |  | 0.77 (0.37, 1.59) |  |
| Out of work for 1 year or more | -- |  | 1.39 (0.52, 3.72) |  | 1.42 (0.51, 4.00) |  | 1.10 (0.39, 3.09) |  |
| Out of work for < 1 year | -- |  | 3.72 (1.01, 13.68)* |  | 3.57 (0.97, 13.15) |  | 5.29 (1.49, 18.76)* |  |
| Out of the work force (includes homemaker, a student, retired, unable to work) | -- |  | 3.86 (2.08, 7.17)* |  | 4.21 (2.25, 7.86)* |  | 3.74 (1.98, 7.07)* |  |
| **Education**  **(ref: College graduate)** |  | -- |  | 0.1317 |  | 0.0809 |  | 0.101 |
| Did not complete high school | -- |  | 2.43 (1.01, 5.85)* |  | 2.16 (1.01, 4.66)* |  | 2.28 (1.03, 5.06)* |  |
| High school graduate | -- |  | 1.04 (0.54, 2.01) |  | 1.03 (0.52, 2.05) |  | 1.24 (0.58, 2.66) |  |
| Some college or technical school | -- |  | 0.85 (0.46, 1.60) |  | 0.84 (0.47, 1.50) |  | 0.94 (0.48, 1.85) |  |
| **Income**  **(ref: <$15,000)** |  | -- |  | <.0001 |  | <.0001 |  | 0.0015 |
| $15,000-<$25,000 | -- |  | 0.75 (0.35, 1.61) |  | 0.8 (0.38, 1.70) |  | 0.92 (0.40, 2.12) |  |
| $25,000-<$35,000 | -- |  | 0.99 (0.43, 2.28) |  | 0.81 (0.37, 1.78) |  | 0.82 (0.33, 2.04) |  |
| $35,000-<$50,000 | -- |  | 0.18 (0.08, 0.42)* |  | 0.21 (0.10, 0.47)* |  | 0.3 (0.12, 0.74)* |  |
| $50,000 or more | -- |  | 0.16 (0.08, 0.35)* |  | 0.18 (0.08, 0.37)* |  | 0.29 (0.13, 0.66)* |  |
| **Health Insurance**  **(ref: No)** |  | -- |  | 0.3402 |  | 0.4226 |  | 0.3367 |
| Yes | -- |  | 2.73 (0.71, 10.56) |  | 2.43 (0.63, 9.35) |  | 2.5 (0.73, 8.57) |  |
| **Personal Doctor**  **(ref: No)** |  | -- |  | <.0001 |  | <.0001 |  | <.0001 |
| Yes, only one | -- |  | 0.93 (0.43, 2.01) |  | 0.66 (0.32, 1.34) |  | 0.78 (0.37, 1.65) |  |
| More than one | -- |  | 3.14 (1.39, 7.06)* |  | 2.34 (1.09, 5.02)* |  | 2.82 (1.29, 6.15)* |  |
| **Caregiving Relationship**  **(ref: Non-relative/Family Friend)** |  | -- |  | -- |  | 0.0226 |  | 0.0293 |
| Child | -- |  | -- |  | 1.78 (0.80, 3.96) |  | 1.98 (0.84, 4.69) |  |
| Other relative | -- |  | -- |  | 0.60 (0.30, 1.20) |  | 0.65 (0.32, 1.35) |  |
| Parent/Parent in law | -- |  | -- |  | 0.99 (0.51, 1.92) |  | 1.25 (0.60, 2.62) |  |
| Spouse/Live-In partner | -- |  | -- |  | 1.40 (0.59, 3.34) |  | 1.75 (0.72, 4.24) |  |
| **Caregiving Hours**  **(ref: Up to 8 hours/week)** |  | -- |  | -- |  | 0.2849 |  | 0.2168 |
| 9 to 19 hours/week | -- |  | -- |  | 0.70 (0.37, 1.33) |  | 0.73 (0.36, 1.49) |  |
| 20 to 39 hours/week | -- |  | -- |  | 0.71 (0.38, 1.34) |  | 0.53 (0.28, 1.02) |  |
| 40 hours or more/week | -- |  | -- |  | 1.18 (0.68, 2.03) |  | 1.05 (0.60, 1.84) |  |
| **Mental Health**  **(ref: 0 days)** |  | -- |  | -- |  | -- |  | <.0001 |
| 1-13 days | -- |  | -- |  | -- |  | 1.88 (0.96, 3.66) |  |
| 14+ days | -- |  | -- |  | -- |  | 8.67 (4.58, 16.41)* |  |

1. Model includes rural/urban status only. [↑](#footnote-ref-1)
2. Model covariates include rural/urban status and sociodemographic factors (age, sex, race/ethnicity, household size, employment, education, income, health insurance, personal doctor). [↑](#footnote-ref-2)
3. Model covariates include rural/urban status, sociodemographic factors (age, sex, race/ethnicity, household size, employment, education, income, health insurance, personal doctor), and caregiving factors (caregiving relationship, caregiving hours). [↑](#footnote-ref-3)
4. Model covariates include rural/urban status, sociodemographic factors (age, sex, race/ethnicity, household size, employment, education, income, health insurance, personal doctor), caregiving factors (caregiving relationship, caregiving hours), and mental health. [↑](#footnote-ref-4)
5. For a multinomial logistic model, the overall Chi-squared test p value for a variable (i.e., race, sex education) will be the same for a variable comparing 1-13 poor physical health days vs 0 days (S6 Table) and comparing 14+ poor physical health days vs 0 days (S7 Table). [↑](#footnote-ref-5)
6. An asterisk (*) indicates that a category is statistically significant (p-value<0.05) from the reference category in terms of the outcome. [↑](#footnote-ref-6)
